# Designing bimetallic lanthanide complexes with composition controlled white-light luminescence for advanced optoelectronics

**DOI:** 10.1039/d6ra07242a

**Published:** 2026-09-29

**Authors:** Bhawna Rathee, Vaishnavi Lather, Rajesh Kumar

**Affiliations:** a University Institute of Engineering and Technology, Maharshi Dayanand University Rohtak - 124001 India lather_rajesh@yahoo.com bhawnarathee7@gmail.com; b Shri Guru Ram Rai Institute of Medical and Health Sciences Dehradun - 248001 India vaishnavilather990@gmail.com

## Abstract

Luminescent complexes containing Tb^3+^ and Sm^3+^ ions were prepared through a solution precipitation method, using ligand L and bathophenanthroline (batho) as an auxiliary ligand. Systematically adjusting the Tb^3+^ to Sm^3+^ ratio enabled precise tailoring of the emission characteristics, resulting in emission profiles that approached white light. The elemental analysis verified the elemental composition. Scanning electron microscopy (SEM) and powder X-ray diffraction (PXRD) confirmed the nanoscale morphology and crystalline structure of the complexes. Fourier-transform infrared (FT-IR) spectroscopy revealed details of the coordination environment, indicating that the ligand L adopts a zwitterionic form upon complexation. Thermogravimetric (TG) and differential thermogravimetric (DTG) analyses showed that the complexes are thermally stable up to 255 °C. Optical band gap analysis confirmed semiconducting behavior. Photoluminescence measurements showed tunable emission features, with the optimal composition (Tb_0.65_ Sm_0.35_) producing white-light emission marked by CIE coordinates of (0.337, 0.371) and a correlated color temperature of 5346.10. Judd–Ofelt analysis (*Ω*_2_ > *Ω*_4_ > *Ω*_6_) indicated notable ligand field asymmetry and rigidity, resulting from cooperative energy transfer between Tb^3+^ and Sm^3+^ ions. Overall, these results suggest that Tb^3+^/Sm^3+^ complexes are strong candidates for multifunctional luminescent materials in optoelectronic and photonic applications.

## Abstract 2

Luminescent complexes containing Tb^3+^ and Sm^3+^ ions were prepared through a solution precipitation method, using ligand L and bathophenanthroline (batho) as an auxiliary ligand.

## Graphical abstract

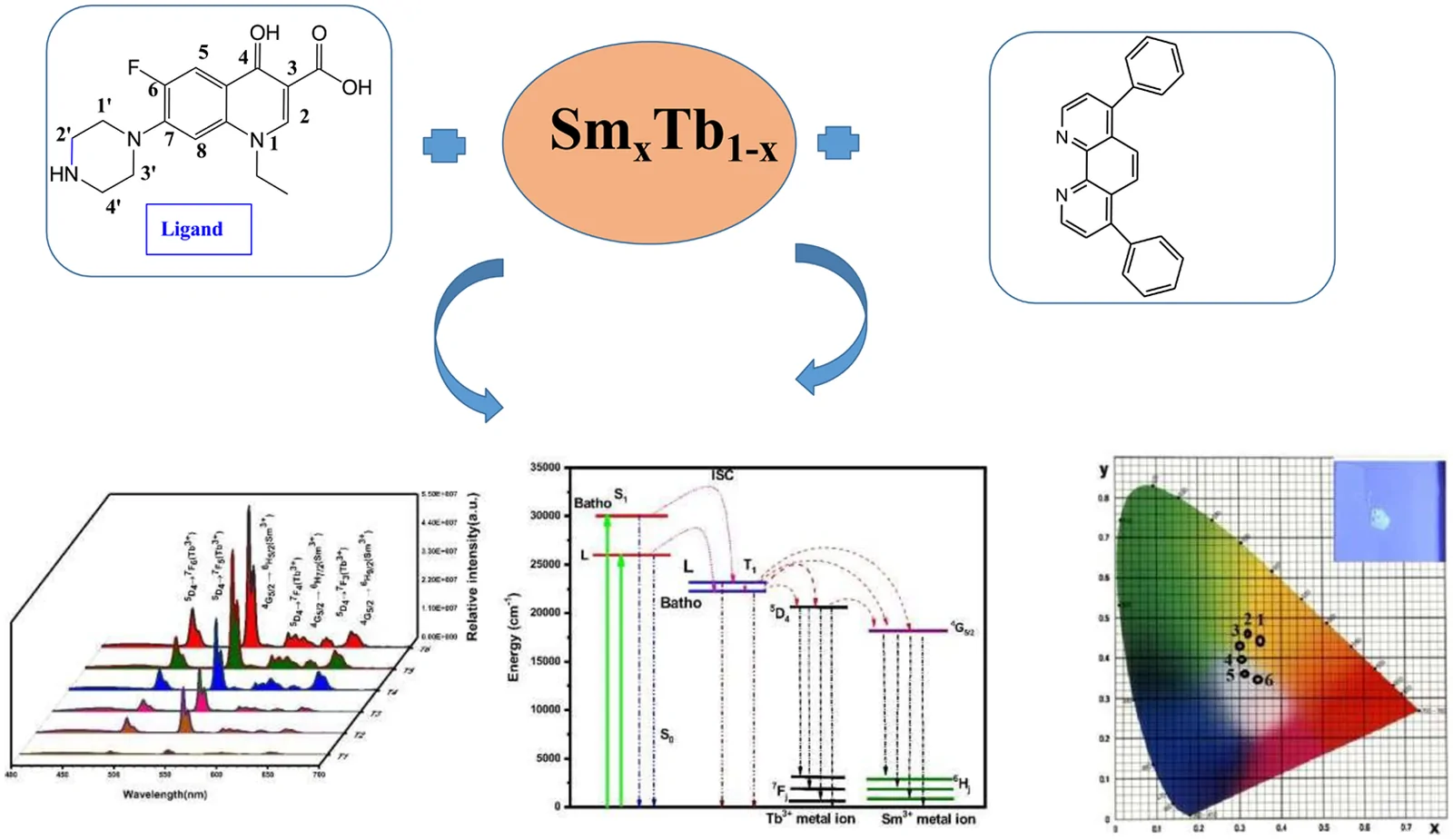

## 1 Introduction

Over recent decades, luminescent rare-earth ions have attracted increasing interest due to their outstanding optical properties and diverse emission capabilities.^1^ These rare-earth-based luminescent materials are widely used in display technology, anti-counterfeiting systems, biosensors, fiber amplifiers, lasers, optical thermometry, and solid-state lighting.^2,3^ Their importance is especially notable in the development of white-light LEDs (WLEDs) and color-tunable phosphors.^4,5^ Solid-state white LEDs have become key lighting sources in the twenty-first century, known for their durability, high efficiency, and environmental stability. Their rapid growth is driven by increasing global demand for ambient lighting amid rising energy consumption. Besides general illumination, white-light emitting diodes (WLEDs) are widely used in colorful displays, temperature sensing, and biotechnology, fuelling research into multicolor emission.^6,7^ This is usually created by mixing rare-earth ions with an organic ligand, which allows emission tuning through mixing ratios.^8,9^ Mixing enables various lighting effects through multiple energy-transfer pathways, resulting in captivating multicolor emission. As a result, systems based on rare-earth ions continue to attract significant research interest, driven by their potential to meet the demand for stable, energy-efficient, and color-accurate optoelectronics (Scheme 1).^10,11^

**Scheme 1:**
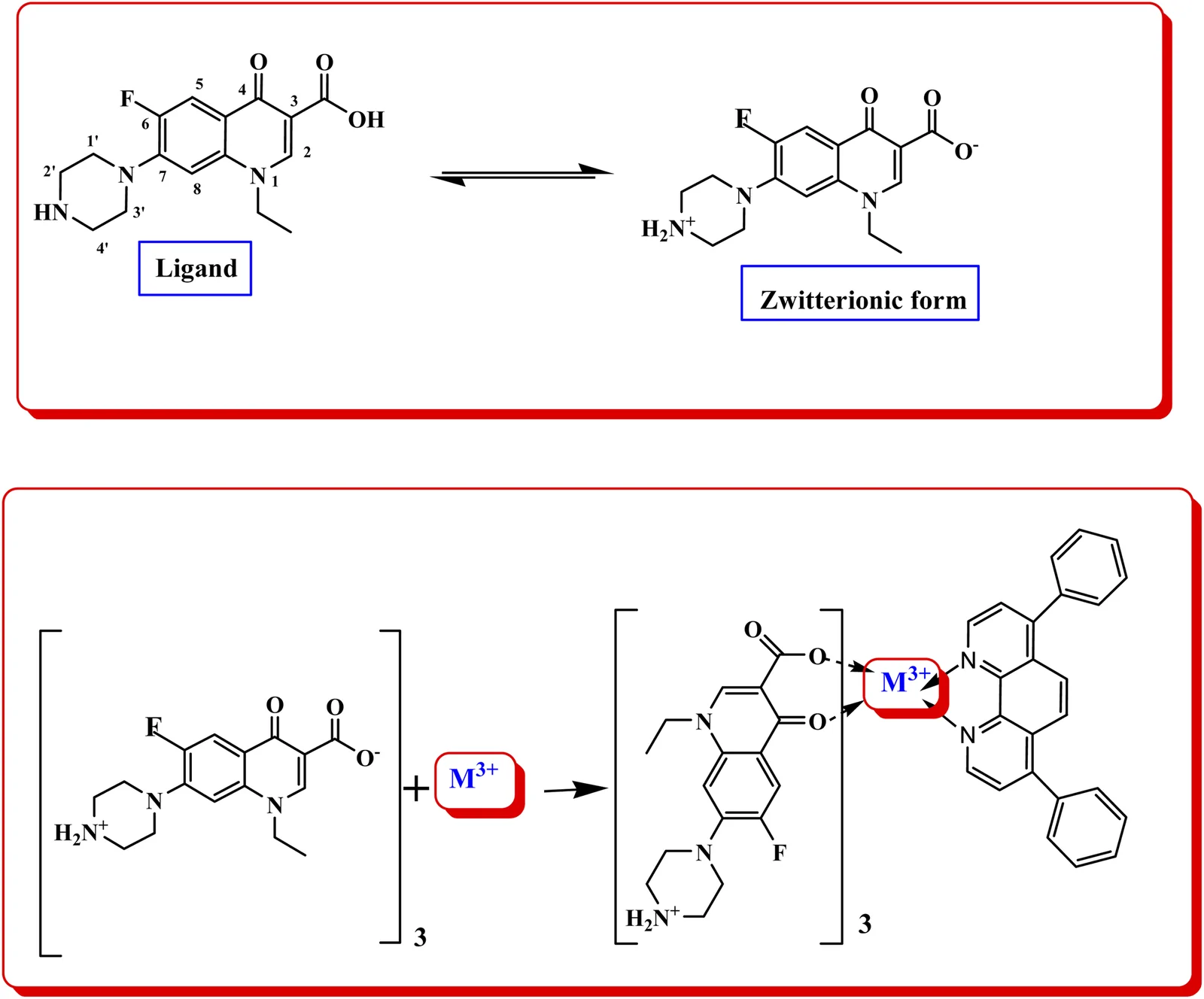
Proposed molecular structure of complexes.

Recent advances in luminescent coordination complexes featuring trivalent terbium (Tb^3+^) and samarium (Sm^3+^) ions have attracted considerable interest.^4,12^ The unique spectral features, increased emission intensity, and effective energy transfer from Tb^3+^ to Sm^3+^ make them excellent candidates for color-tunable luminescence. In coordination complexes, these ions are often sensitized by organic ligands functioning as antennas, which capture incident light and relay energy to the lanthanide ion through processes such as Dexter or Förster energy transfer. The emitted light depends strongly on factors such as the ligand environment and the energy-transfer mechanism. These complexes are notable for their ability to produce tunable emission and near-white light. By adjusting the molar ratio of lanthanide ions in an organic ligand, researchers can finely tune emission colors, quantum yields, and other photophysical properties.

To meet the rising demand for advanced optoelectronic materials, we developed a series of innovative organo-lanthanide complexes. These were synthesized using 1-ethyl-6-fluoro-1,4-dihydroxy-4-oxo-7-(1-piperazinyl)-3-quinoline carboxylic acid (L) as the primary antenna ligand. Selected for its strong carboxylate binding and extended π-conjugation, this ligand promotes efficient energy transfer to Sm^3+^ and Tb^3+^ ions. Fluorine substitution enhances radiative efficiency by altering the electron density and reducing vibrational quenching. Additionally, batho was introduced as an ancillary ligand to increase luminescence intensity by suppressing nonradiative pathways and stabilizing excited states. To attain white-light emission, Sm(iii) and Tb(iii) ions were co-doped at various molar ratios, allowing precise tuning of their emission properties. The semiconducting, lasing, and luminescent characteristics of these complexes were extensively examined using techniques such as elemental analysis, SEM, IR spectroscopy, X-ray diffraction, TGA/DTG, voltammetry, optical and photoluminescence studies confirmed their potential in optoelectronic application. The emission spectra facilitated the assessment of radiative and chromaticity parameters. Judd–Ofelt (JO) analysis of NIR absorption spectra provides detailed information on their local geometry and bonding environments. These complexes are useful beyond lighting applications, notably in forensic science for visualizing latent fingerprints (LFPs) under ultraviolet light and for anti-counterfeiting applications to detect fake certificates and RFID tags. Overall, this research presents a new method for producing white-light emission by precisely mixing lanthanide ions, providing a flexible approach to color-tunable luminescence. In this study to report the dual use of these complexes in both optoelectronic and forensic applications.

## 2 Experimental section

### 2.1 Materials and methods

All chemicals used in this research work are of excellent analytical grade and were purchased from Sigma-Aldrich. They are used exactly as intended and without any additional purification.

The CHN elemental analysis of the complexes was performed with a Euro EA analyzer. SEM studies were carried out on a Jeol JSM-6510 equipped with an AMETEK EDAX for recording electron-dispersive X-ray spectra to confirm the composition of complexes. Powder XRD patterns were recorded on a Rigaku Ultima IV diffractometer at 40 kV and 40 mA, with a scan rate of 2° min^−1^ from 10° to 70° using Cu Kα radiation. A Bruker FTIR spectrometer was used to record FTIR spectra within the 400–4000 cm^−1^ range. TGA and DTG analyses were conducted with a PerkinElmer STA-6000 at a heating rate of 10 °C min^−1^ under a nitrogen atmosphere. PL spectra and lifetimes were measured with a Hitachi F-7000 fluorescence spectrophotometer using FL Solution software. For PL measurements, the complexes were dissolved in DMSO at a concentration of 10^−3^ M. The prepared solutions were transferred to quartz cuvettes, and the spectra were recorded under identical experimental conditions using a slit width of 1 nm to ensure reliable comparison of the emission characteristics of the complexes. UV/vis absorption spectra were recorded with a Shimadzu UV-3600 Plus spectrophotometer, which also measured the diffuse reflectance spectra of the ligand and complexes in the 200–800 nm range, using barium sulfate as the reference. CIE color coordinates of T1–T6 complexes were determined using MATLAB software. Using the fitting application of Origin software on experimental data, the optical energy band gap, FWHM, and Urbach energy were computed.

### 2.2 Method of synthesis

Six ternary complexes (T1–T6) were systematically created with increasing Tb^3+^ and decreasing Sm^3+^ content, covering the range from Tb_0_._50_Sm_0_._50_ to Tb_0_._75_Sm_0_._25_. They were synthesized using a solution-precipitation method under controlled conditions to ensure consistency. For preparing the representative T1 complex, a 3 mmol ethanolic solution of the primary ligand (L) was mixed with an aqueous solution containing Sm^3+^ and Tb^3+^ ions in the desired ratio, with continuous magnetic stirring. After 30 minutes, an ethanolic bathophenanthroline solution was added. The molar ratio of metal to ligand to bathophenanthroline (M : L : batho) was kept at 1 : 3 : 1. The pH was adjusted to 7 with sodium hydroxide, and stirring continued for 4–5 hours at 45 °C. The off-white precipitate was then filtered, washed with water and ethanol, and dried at room temperature.^13,14^

The remaining complexes (T2–T6) were synthesized similarly, with only minor variations in the concentrations of Sm^3+^ and Tb^3+^. This careful tuning of precursor ratios enabled controlled changes in Tb/Sm content across the series, facilitating a detailed study of how composition influences the structural and photophysical properties of the complexes and aiding the attainment of white light. The T6 complex was identified as having emission properties that position it exactly in the white region of the chromaticity diagram. This optimal combination of Tb^3+^ and Sm^3+^ contributions make T6 the most suitable complex for producing pure white luminescence. Therefore, its structural, morphological, and photophysical characteristics were thoroughly analyzed. Generally, complexes are represented by T1 [Tb_0.40_Sm_0.60_], T2 [Tb_0.45_Sm_0.55_], T3 [Tb_0.50_Sm_0.50_], T4 [Tb_0.55_Sm_0.45_], T5 [Tb_0.60_Sm_0.40_], T6 [Tb_0.65_Sm_0.35_].

## 3 Results and discussion

### 3.1 General characterization

Elemental analysis was performed to confirm the stoichiometry of carbon, hydrogen, and nitrogen in the synthesized complexes. The experimental results (Table 1) closely matched the theoretical percentages derived from the proposed chemical formulas for T1–T6, confirming the structural accuracy of the assignments.

**Table 1:** Analytical characterization results of T1–T6 complexes

Complexes	C (%) found (cal.)	H (%) found (cal.)	N (%) found (cal.)
T1	59.85 (59.87)	04.80 (04.85)	10.65 (10.67)
T2	59.81 (59.84)	04.81 (04.84)	10.63 (10.66)
T3	59.78 (59.82)	04.80 (04.84)	10.64 (10.66)
T4	59.78 (59.80)	04.79 (04.84)	10.62 (10.65)
T5	59.75 (59.77)	04.80 (04.84)	10.62 (10.65)
T6	59.74 (59.76)	04.79 (04.84)	10.63 (10.65)

The microstructure and surface features of a representative T6 complex were analyzed using scanning electron microscopy (SEM). As shown in Fig. 1(a), the SEM image demonstrates that T6 mainly has a spherical shape, suggesting consistent particle formation. The distribution of metal ions was investigated using the EDAX method. The EDAX spectra verified the presence of elements C, O, F, N, Tb, and Sm as shown in Fig. 1(b). The purity of the complex was confirmed by the lack of spectrum peaks related to impurities.

**Fig. 1:**
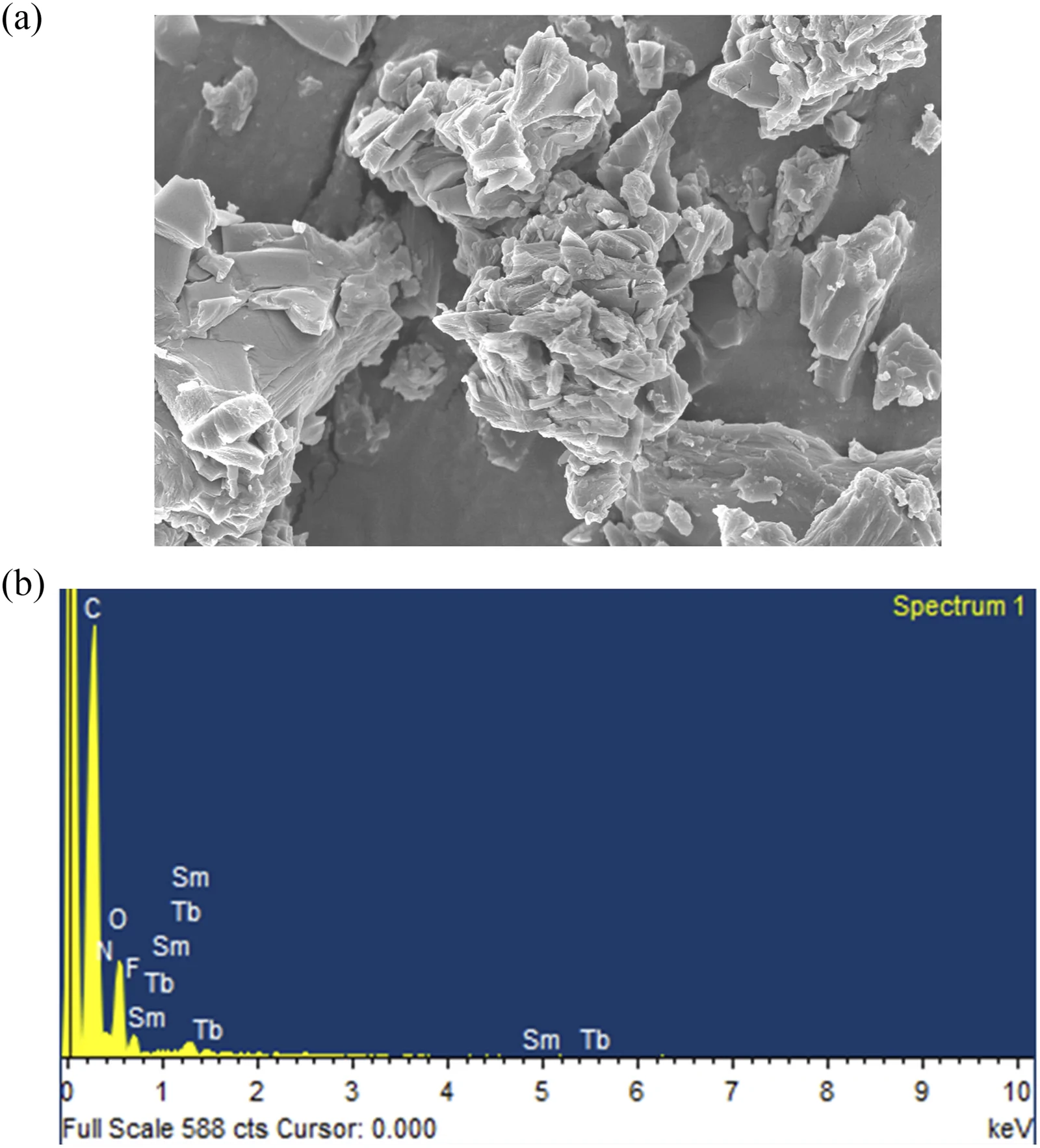
(a). SEM micrograph of complex T6. (b). EDAX spectra of T6 complex.

### 3.2 X-ray diffraction analysis

Powder X-ray diffraction (PXRD) analysis of the T6 complex was performed at room temperature over the 10–70° (2*θ*) range, with a scan rate of 3° min^−1^. The diffraction pattern showed distinct, sharp peaks at specific 2*θ* angles, indicating a crystalline structure. The average crystalline grain size was calculated using the Scherrer equation.1
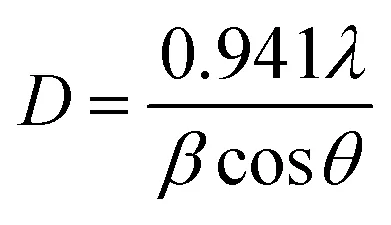
Here, *β* represents the FWHM (full width at half maximum), *λ* is 0.154056 nm, *θ* indicates the position of XRD signals, and *D* is the average crystallite size. The crystallite size of the T6 complex is 8.38 nm.^15^ The presence of distinct sharp peaks in the XRD (Fig. 2) confirms the crystalline nature of the complex. This observation is also supported by the calculated crystallinity index of 82.34%. The crystallinity index of the complex is calculated as the ratio of the integrated area of the crystalline peaks to the integrated area under all XRD peaks.

**Fig. 2:**
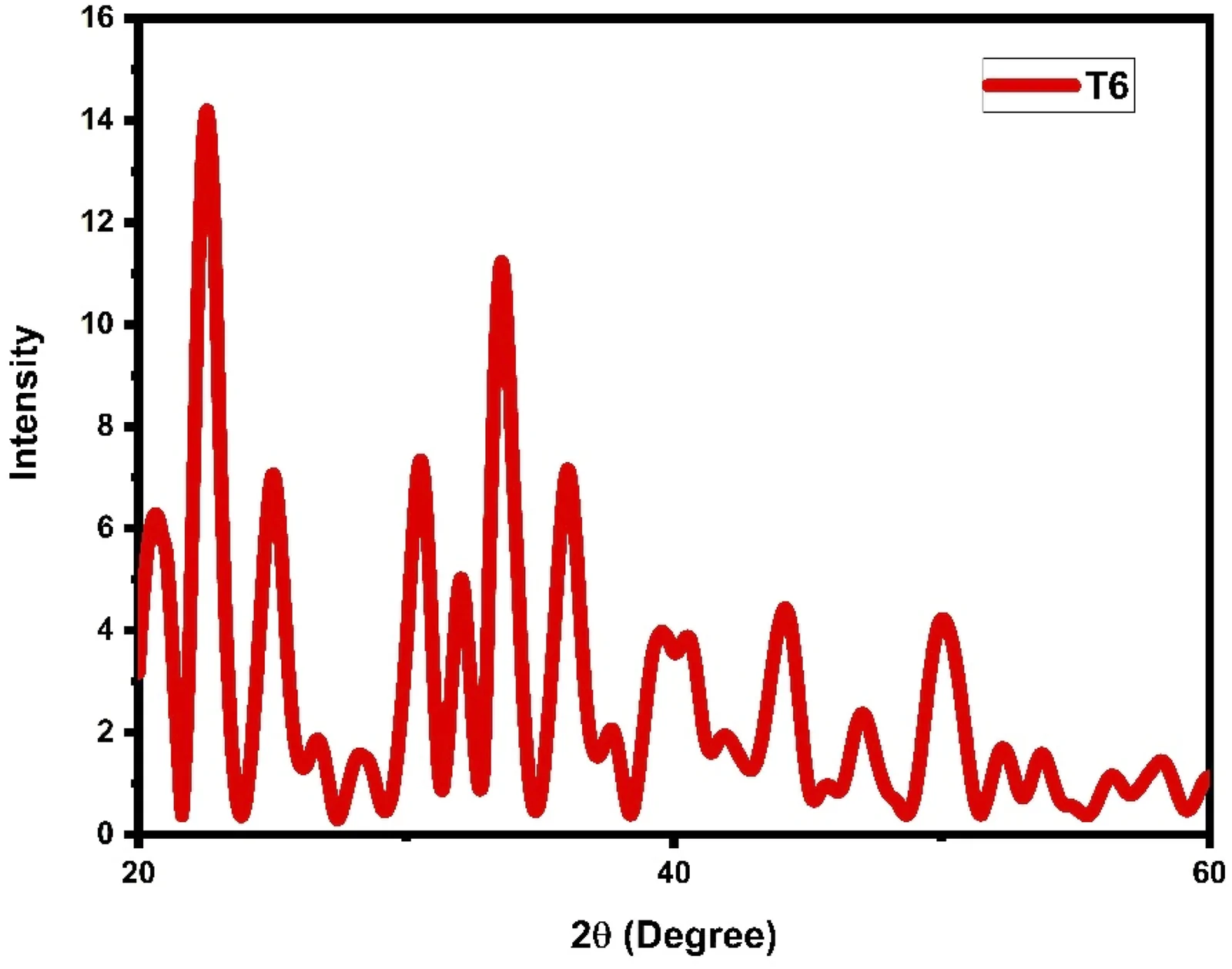
XRD pattern of T6 exhibiting its crystalline nature.

### 3.3 FT-IR spectral studies

The nature of bonding between the ligand and metal ions was clarified through comparative FTIR spectral analysis of the free ligand (L) and the T6 complex (Fig. 3). In the spectrum of the free ligand, a broad absorption at 3439 cm^−1^ corresponds to O–H and N–H stretching vibrations. When complexation, this band shifts to 3410 cm^−1^, indicating O–H stretching from coordinated water molecules. The prominent ketonic C=O stretch at 1630 cm^−1^ in the ligand shifts to 1620 cm^−1^ in the complex, supporting chelation and suggesting that the carbonyl oxygen participates in coordination.^16,17^

**Fig. 3:**
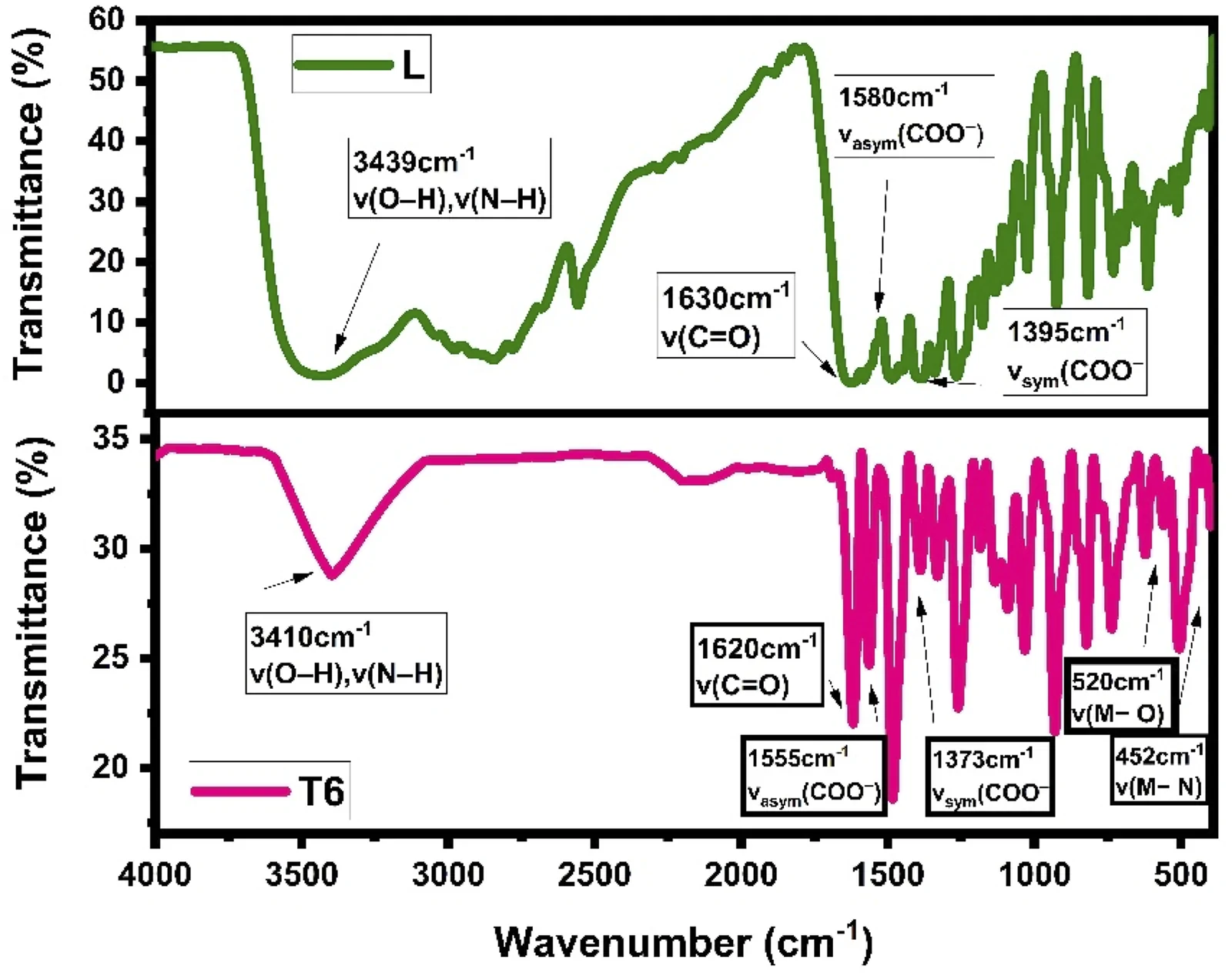
Prominent IR spectral bands of L and T6.

The multiple absorption bands with varying intensities observed in the 2800–2700 cm^−1^ and 2500–2400 cm^−1^ regions strongly indicate nitrogen quaternization within the piperazinyl group, confirming that L exists as a zwitterionic state.^18–20^ This is indicated by asymmetric (1580 cm^−1^) and symmetric (1395 cm^−1^) COO^−^ stretching vibrations. In the complex, these bands shift to 1555 cm^−1^ and 1373 cm^−1^, respectively, implying coordination *via* the carboxylate oxygen. The difference between the asymmetric and symmetric stretchings (Δ*ν* = 183 cm^−1^) confirms that the carboxylate group binds in a monodentate manner.^21,22^

Moreover, the FTIR spectrum of the T6 complex shows extra bands in the 450–550 cm^−1^ range, attributed to M–O and M–N stretching vibrations. These observations confirm that the ligand binds to the metal through oxygen atoms in the carboxylate and carbonyl groups, while the auxiliary ligands are probably attached *via* nitrogen atoms.

### 3.4 Thermal analysis

For any luminescent material intended for display applications, thermal stability is a critical requirement. The thermal behavior of the T6 complex was examined using TGA and DTA analyses over the range of 30–900 °C, as shown in Fig. 4. The decomposition occurs in three distinct stages. The first step, up to 88.88 °C, shows an 8% mass loss, corresponding to the release of seven water molecules (calculated 8.10%). No weight change is observed until 255 °C, confirming that the complex remains stable up to this temperature. The second stage involves a 30.12% mass loss up to 460 °C, attributed to the elimination of the ancillary ligand and one L molecule (calculated 32.80%). In the final stage, partial degradation of another L molecule results in a further 16.96% mass loss up to 900 °C. At this point, approximately 36.76% of the original mass remains, consisting mainly of metal oxide and carbonaceous residue from incomplete L decomposition.^23^ The DTA profile supports these sequential degradation steps. Overall, the thermal analysis highlights the compound's stability and reinforces its suitability for display applications.^24^

**Fig. 4:**
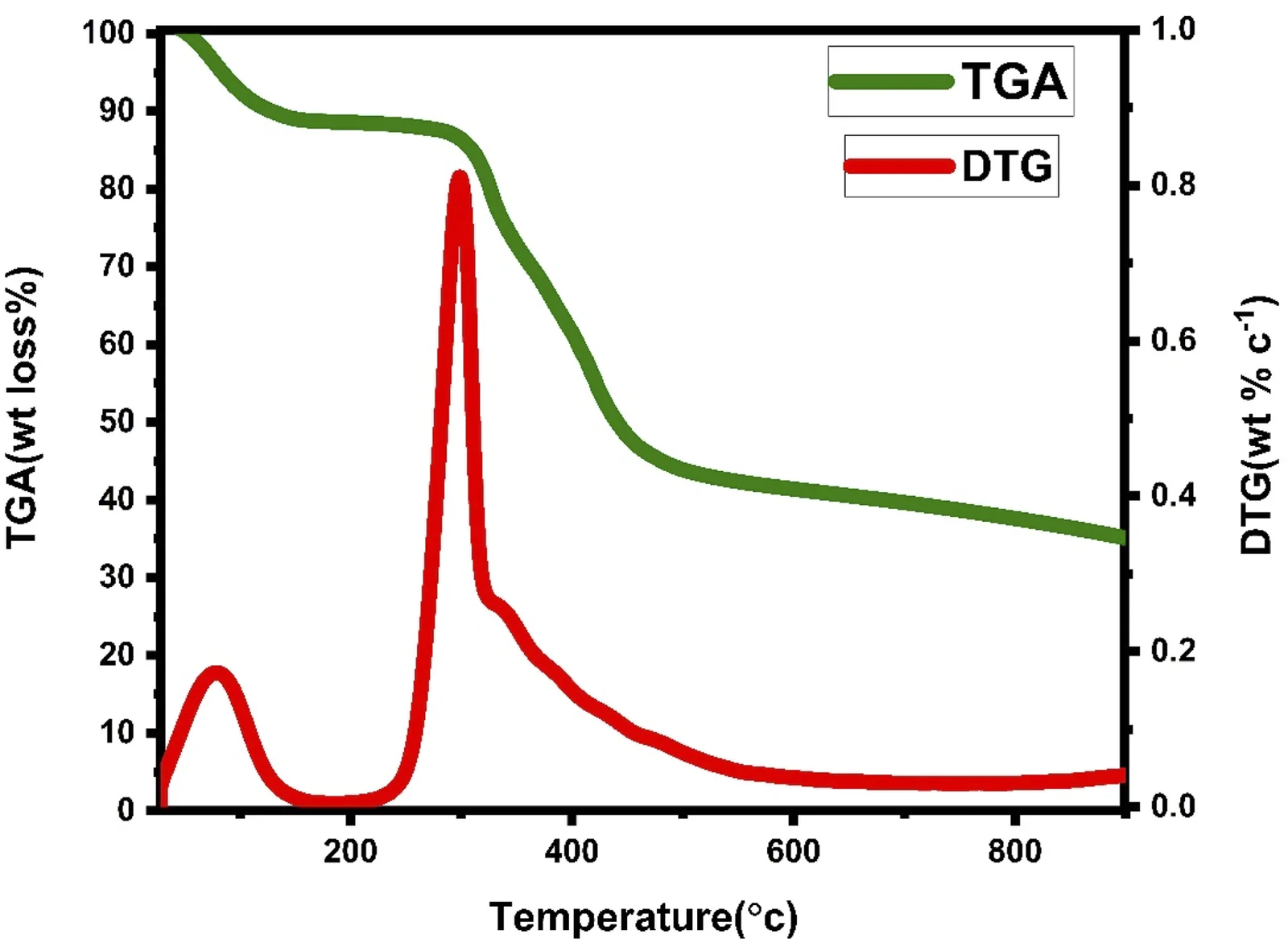
Thermal analysis of T6 by means of TGA and DTG under inert atmosphere.

### 3.5 Photoluminescence spectra

Incorporating multiple lanthanide ions such as Sm^3+^ and Tb^3+^ into a single coordination complex with an organic ligand enables tunable photoluminescent activity, multicolor emission, and energy transfer effects.^9,25^ The characteristic red emission of Sm^3+^ and green emission of Tb^3+^ can be effectively combined with the ligand, and the appropriate mixing of these complementary emission components can generate white-light emission. Thus, the relative contributions of Sm^3+^ and Tb^3+^ emissions play an important role in controlling the overall emission color and provide a potential strategy for the development of white-light-emitting luminescent materials. To evaluate these properties, photoluminescence (PL) measurements of excitation and emission were conducted in both solution and solid phase. The excitation spectra at 548 nm emission wavelengths show a prominent band around 370 nm in both states. Fig. 5(a) and (b) present the excitation spectra of all complexes in both solid and solution phases. This band mainly arises from π–π* transitions within the aromatic ligand framework, with potential contributions from ligand-to-metal charge transfer (LMCT) or metal-to-ligand charge transfer (MLCT) transitions that enhance sensitization of lanthanide ions.^26^ The ligand acts as an antenna by absorbing the excitation energy and sensitizing the lanthanide centres. Following ligand-to-lanthanide energy transfer, the excited Tb^3+^ centre can serve as an energy donor to Sm^3+^, provided that the relevant energy levels are suitably matched.

**Fig. 5:**
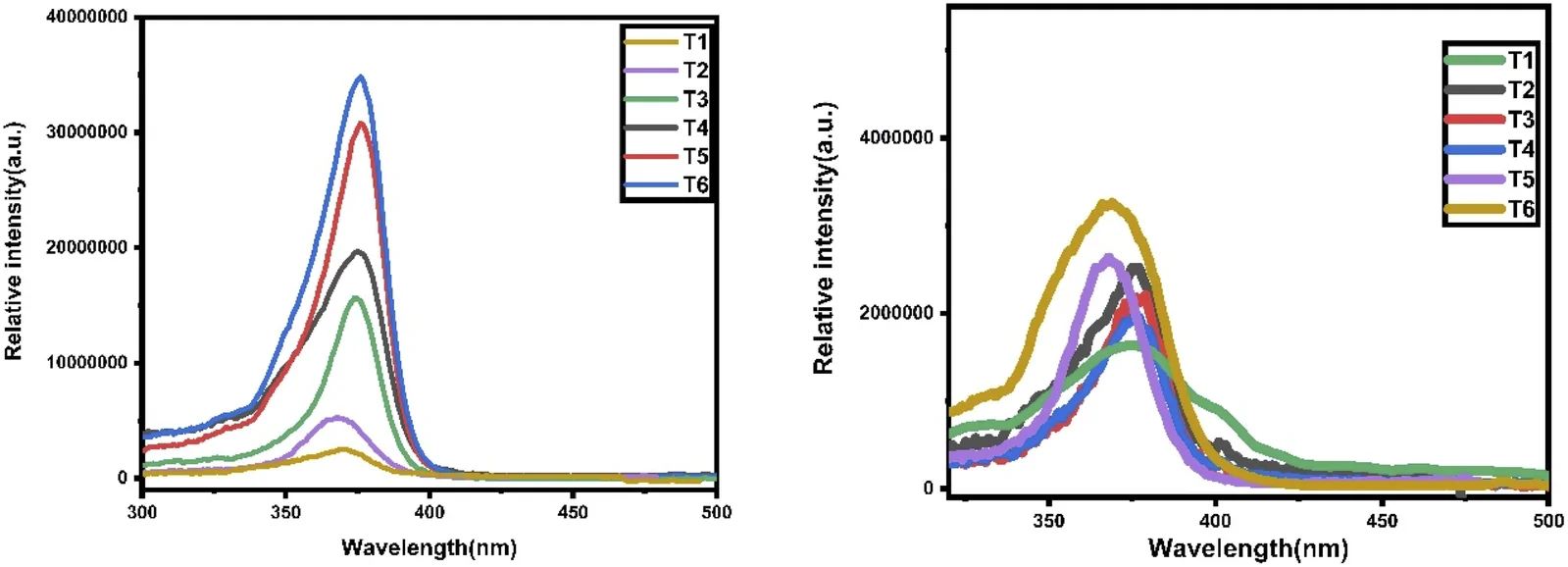
Photoluminescent excitation spectra of complexes T1–T6 (a) in solution phase (b) in solid phase at emission 548 nm.

Excitation at 370 nm revealed characteristic emissions from both Sm^3+^ and Tb^3+^ ions. Fig. 6(a) and (b) illustrate the emission spectra in both phases. In solution phase (DMSO), Sm^3+^ showed emission bands at 560, 602, and 650 nm, linked to the ^4^G_5/2_ → ^6^H_5/2_, ^4^G_5/2_ → ^6^H_7/2_, and ^4^G_5/2_ → ^6^H_9/2_ transitions.^27^ Tb^3+^ exhibited peaks at 491, 548, 585 and 620 nm, corresponding to the ^5^D_4_ → ^7^F_6_, ^5^D_4_ → ^7^F_5_, ^5^D_4_ → ^7^F_4_ and ^5^D_4_ → ^7^F_3_ transitions.^28^ In solid state, Sm^3+^ emitted at 562, 605, and 650 nm, while Tb^3+^ transitions appeared at 488, 548, 585 and 617 nm, matching the ^4^G_5/2_ → ^6^H_5/2_, ^4^G_5/2_ → ^6^H_7/2_, ^4^G_5/2_ → ^6^H_9/2_, ^5^D_4_ → ^7^F_6_, ^5^D_4_ → ^7^F_5_, ^5^D_4_ → ^7^F_4_ and ^5^D_4_ → ^7^F_3_ transitions, respectively. The electric dipole transitions (^5^D_4_ → ^7^F_5_ and ^4^G_5/2_ → ^6^H_9/2_) proved to be highly sensitive to the local environment surrounding the central lanthanide ion, while the magnetic dipole transitions (^5^D_4_ → ^7^F_6_ and ^4^G_5/2_ → ^6^H_5/2_) remained mostly unaffected. The ^4^G_5/2_ → ^6^H_7/2_ transition (Δ*J* = 1) showed a mixed electric and magnetic dipole character, with a dominant electric component.^29^ The photoluminescence intensity shows a clear dependence on the Tb^3+^ concentration. As the Tb^3+^ content increases, the intensity of the characteristic Tb^3+^ emission bands also increases. This enhancement can be attributed to the increasing population of Tb^3+^ emitting centres and the greater contribution of Tb^3+^-centred radiative transitions to the overall emission.

**Fig. 6:**
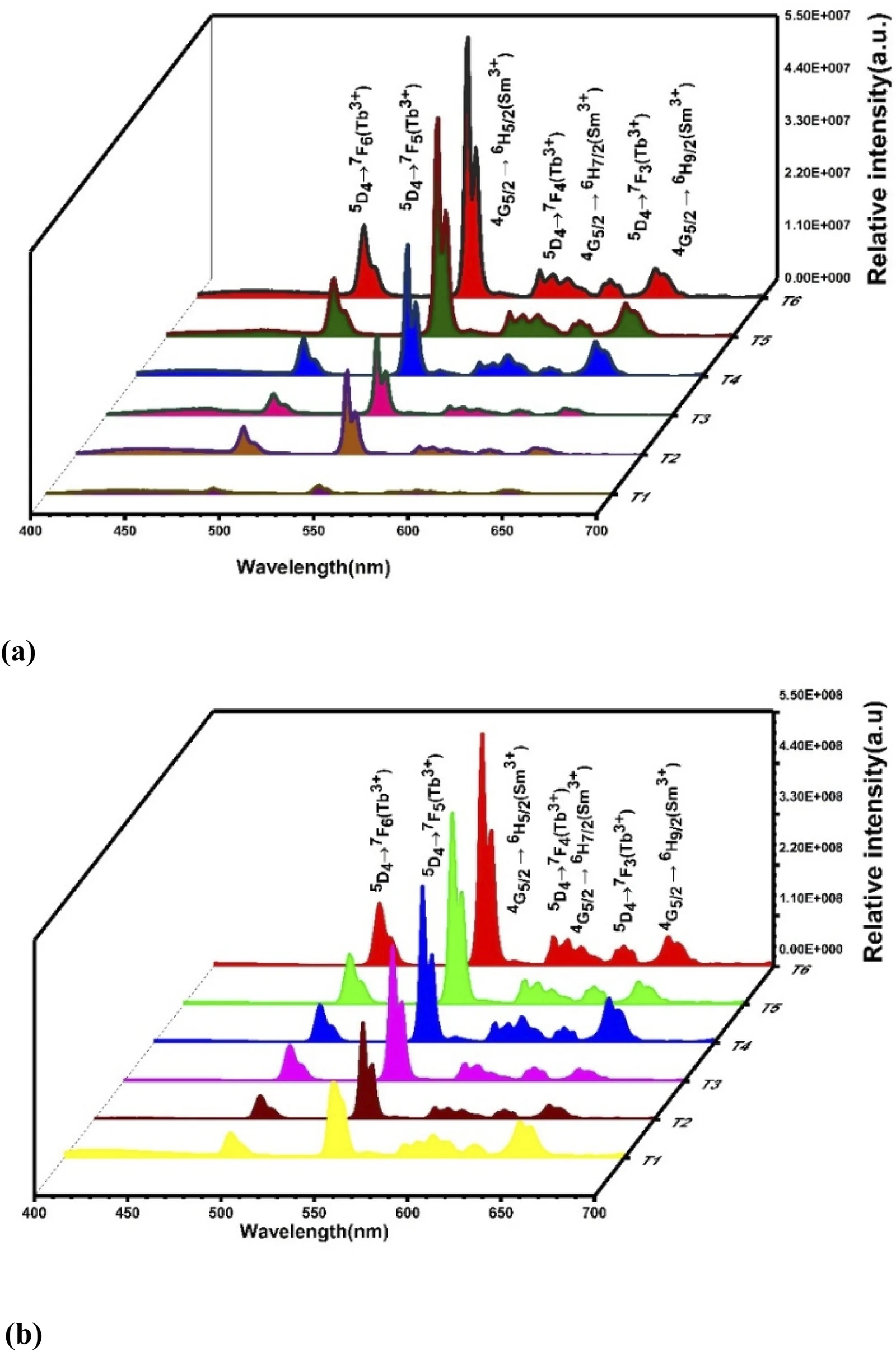
Photoluminescent emission spectra of complexes T1–T6 (a) in solution phase and (b) in solid phase at excitation 370 nm.

In addition, the ligand can sensitize the Tb^3+^ ions through the antenna effect, facilitating the population of their excited states and consequently enhancing the observed emission intensity. The increase in Tb^3+^ concentration also influences the energy-transfer processes within the mixed-lanthanide system. The overall emission of the mixed-lanthanide complexes is governed by the combined effects of ligand sensitization, Tb^3+^ → Sm^3+^ energy transfer, and the relative concentration of the two lanthanide ions. The solid-state emission spectra of the L, recorded at an excitation wavelength of 370 nm, are presented in Fig. S7. The L exhibits a broad emission feature spanning 400–500 nm. In contrast, the emission spectra of the complexes lack these broad sensitizer-derived humps and instead reveal the characteristic sharp transitions of metal ions. A direct comparison of the sensitizer emission with that of complexes T1–T6 clearly indicates the absence of sensitizer-based luminescence in the 400–500 nm range, thereby confirming efficient energy transfer from the sensitizers to the metal ion.

### 3.6 Chromaticity analysis

Chromaticity parameters such as CIE coordinates (*x*, *y*), correlated color temperature (CCT) and color purity (CP) were derived from the emission spectra. MATLAB was used to determine the chromaticity coordinates of the complexes, facilitating the assessment of their photonic characteristics. These coordinates were then plotted on the CIE chromaticity diagram to illustrate the tunable emission behavior (Fig. 7).

**Fig. 7:**
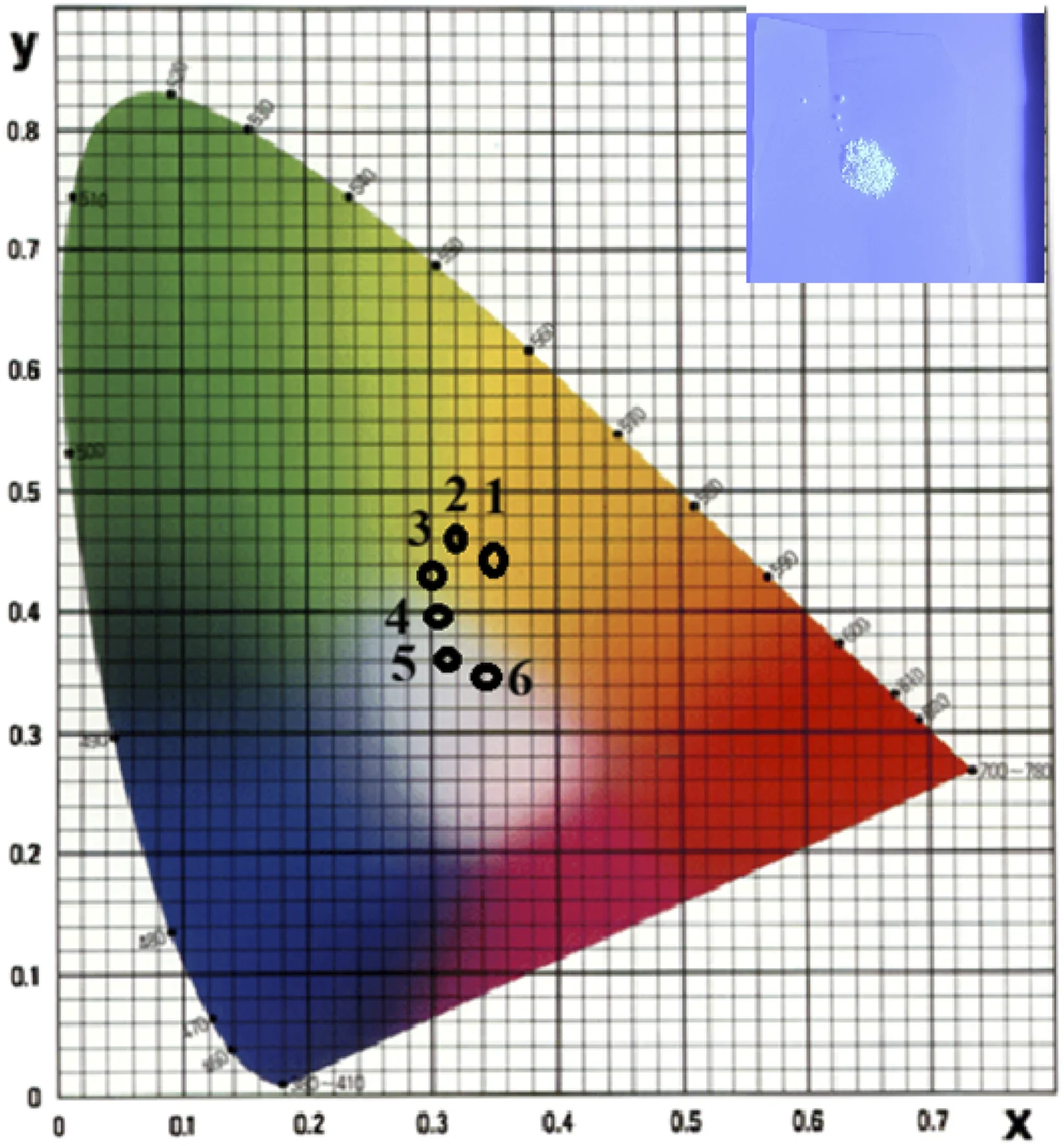
CIE chromaticity coordinates (*x*, *y*) of complexes (T1–T6) with inset showing the T6 under UV illumination.

Complexes T1–T6 have coordinates near the white-light region in the diagram, with T6 clearly within the white zone, indicating a balanced emission profile. The closeness of these coordinates to the white-light region confirms that the complexes can produce near-white emission. Additionally, the emission color can be precisely tuned toward white light by changing the relative concentrations of Sm^3+^ and Tb^3+^ ions.^30,31^ The CCT values for all complexes were determined using the McCamy equation, as shown below.

2 CCT = −437*n*^3^ + 3601*n*^2^ − 686*n* + 5514.31

The chromaticity center is defined as (*x*_c_, *y*_c_) is (0.332, 0.186) within the relation *n* is (*x* − *x*_c_)/(*y* − *y*_c_), where (*x*, *y*) represent the CIE 1931 chromaticity coordinates. Light emissions are classified as neutral between 3200 and 4000 K, warm below 3200 K, and cool above 5000 K based on correlated color temperature (CCT). The investigated complexes emit cold white light, with CCT values ranging from above 5000 K. Moreover, the color purity (CP), an important colorimetric parameter, can be calculated and analyzed from the chromaticity coordinates (*x*, *y*) using the following formula.3
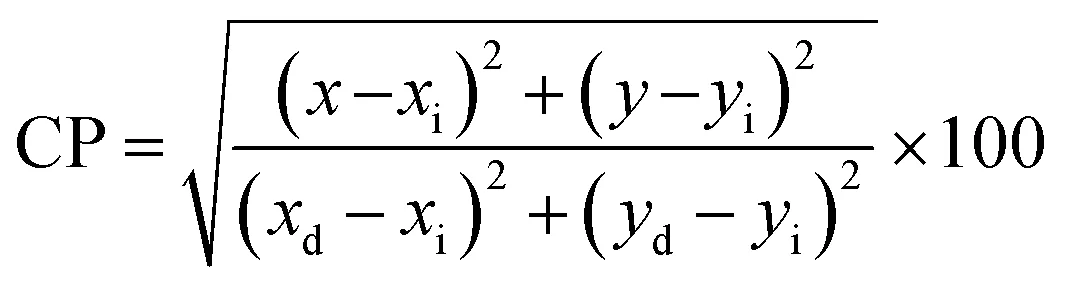


The primary color coordinates are (*x*_d_, *y*_d_) and (*x*_i_ = 0.333, *y*_i_ = 0.333). Owing to their high color purity, these synthetic complexes could be valuable for various laser applications. The equation below was used to compute another pair of chromaticity parameters, *u*′ and *v*′.4
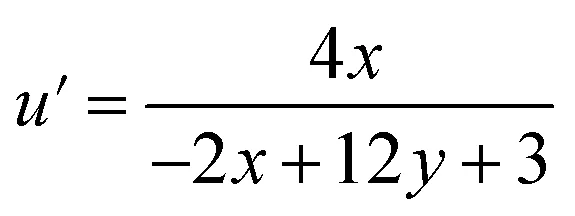
5
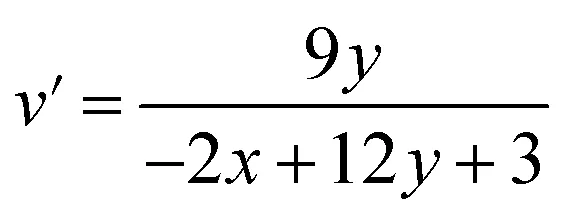


Table 2 summarizes the *u*, *v* chromaticity coordinates, color purity (CP), and correlated color temperature (CCT) for each complex. The emission color characteristics are assessed using the CIE 1931 chromaticity diagram. Coordinates like (0.337, 0.371) with a CCT around 5346.10 K indicate high-quality white light, making these materials suitable for lighting applications.

**Table 2:** Summarizes the chromaticity coordinates and related parameters for T1–T6

Complexes	*x*, *y*	CP (%)	CCT (K)	*u*′*v*′
T1	0.347, 0.458	36.81	5151.37	0.1779, 0.5283
T2	0.321, 0.463	38.07	5792.96	0.1622, 0.5265
T3	0.308, 0.434	30.35	6211.61	0.1622, 0.5144
T4	0.303, 0.417	26.01	6433.92	0.1638, 0.5072
T5	0.324, 0.377	13.53	5828.63	0.1884, 0.4934
T6	0.337, 0.371	11.87	5346.10	0.1988, 0.4926

### 3.7 Diffuse reflectance optical analysis

The optical band gaps (*E*_g_) of complexes T1–T6 were measured using their diffuse reflectance (DR) spectra, with barium sulfate as a reference. Fig. S1 shows the Tauc plots for both the ligand (L) and the complexes. The band gap values were calculated *via* the Tauc relation^32,33^

6 [*αhν*]^2^ = *c*[*hν* − *E*_g_]

where *h* is Planck's constant, *ν* is the incident light's frequency, *α* is the absorption coefficient, and *c* is band tailing constant.

Compared to the ligand, all complexes have lower *E*_g_ values because the metal ions introduce additional electronic states, narrowing the band gap. The *E*_g_ values for the complexes range from 2.8 to 3.0 eV, indicating they are semiconductors. Pratibha *et al.*, however, reported *E*_g_ values of 3.00–3.08 eV for the same ligand in samarium complexes.^22^ Incorporating both Sm^3+^ and Tb^3+^ ions creates new energy levels between the valence and conduction bands, resulting in smaller band gaps (<3.00 eV).^34^ These results, listed in Table 3, suggest these complexes could be useful in semiconductor applications.

**Table 3:** Optical parameters: band gap (*E*_g_), Urbach energy (*U*_e_), and refractive index (*n*) of L and T1–T6

	Bandgap		Urbach energy		Refractive index
	*E*_g_ (ev)	*R* ^2^	*U*_e_ (eV)	*R* ^2^	*n*
L	3.03	0.9966	0.1893	0.9971	2.034
T1	2.86	0.9994	0.155	0.9991	2.070
T2	2.93	0.9939	0.101	0.9805	2.055
T3	2.96	0.9991	0.068	0.9998	2.049
T4	2.98	0.9884	0.066	0.9995	2.044
T5	2.99	0.9972	0.063	0.9973	2.042
T6	3.00	0.9985	0.062	0.9996	2.040

To examine their optical behavior further, the Urbach energy (*U*_e_) was calculated using:7
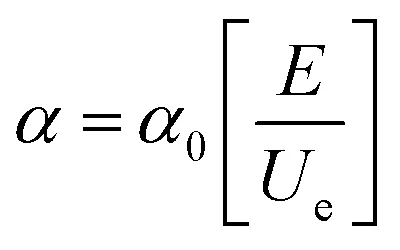
where *α*_0_ is a material-specific constant. *U*_e_ is derived by inverting the graph's slope, as shown in Fig. S2. The complexes have lower *U*_e_ values than the ligand, indicating fewer structural defects and fewer localized states.^35^

Additionally, the refractive index (*n*) was estimated using the empirical formula:8
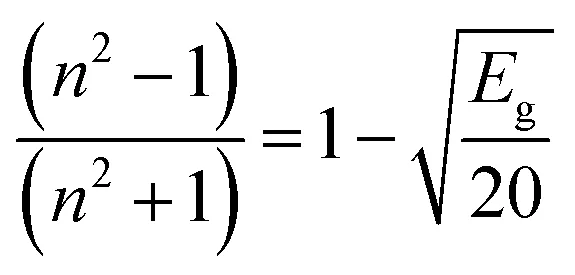


The refractive index values derived from the above equation are shown in Table 3. They meet the criteria for optoelectronic applications, highlighting the potential of these complexes in such technologies.^36,37^

### 3.8 Electrochemical characterization

The electrochemical properties of the T6 complex were explored using cyclic voltammetry (CV) analysis. The current–potential characteristics were measured in dichloromethane (DCM, 10^−3^ M) containing 0.1 M tetraethylammonium perchlorate (TEAP) as the supporting electrolyte over the potential interval of 0 V to +6 V, employing a sweep velocity of 0.1 V s^−1^. The experiment was performed using a standard three-electrode assembly comprising a glassy carbon working electrode, platinum counter electrode, and silver wire reference electrode. The obtained voltammogram for the T6 complex is depicted in Fig. S3, while the inset displays the ferrocene (Fc) reference system employed for calibration.

The oxidation and reduction processes observed in the voltammogram provide important information regarding the frontier molecular orbital (FMO) energy architecture of the complex. The HOMO and LUMO energy levels were estimated using the following equations:

9 *E*_HOMO_ = −[(*E*_0*x*_) + 3.77] eV

10 *E*_LUMO_ = −[*E*_red_ + 3.77] eV

The ferrocene standard displayed a half-wave potential (*E*_1/2_) of 1.03 V. Furthermore, the oxidation and reduction potentials of the T6 complex were observed at 3.714 V and 2.311 V, respectively, corresponding to HOMO and LUMO energy levels of −7.484 eV and −6.081 eV. Based on these values, the electrochemical band gap (*E*_g_) was estimated to be 1.403 eV. The optical band gap estimated from the diffuse reflectance spectrum using the Tauc method was 3.00 eV, which can be associated with the onset of an optically allowed, predominantly ligand-centered π–π* transition. In contrast, the electrochemical band gap estimated from the oxidation and reduction potentials obtained from cyclic voltammetry was 1.40 eV, providing an estimate of the frontier-orbital energy separation. The difference between these values reflects the different physical processes probed by optical spectroscopy and electrochemical measurements; therefore, the two values should not be considered directly equivalent. Rather, the optical and electrochemical measurements provide complementary information about the electronic structure of the material. Furthermore, the observed difference between the electronic bandgap (*E*_g_) obtained from cyclic voltammetry and the optical bandgap can be attributed to the influence of ancillary ligands in the complex. These ligands can modulate the electronic distribution by stabilizing or destabilizing the frontier molecular orbitals, thereby altering the redox potentials. In particular, electron-donating or conjugated auxiliary ligands can raise the HOMO energy level or lower the LUMO energy level, thereby reducing the electrochemical bandgap relative to the optical bandgap. In addition, the incorporation of both Sm^3+^ and Tb^3+^ ions within the same coordination framework can subtly alter the electronic environment surrounding the ligand, leading to modifications in the frontier orbital energies. This behavior highlights the significant role of ligand design in tuning the electronic properties of the corresponding complex, allowing for precise control over its frontier orbital energies and potential photophysical applications.

### 3.9 Absorption spectra

The absorption spectra of the complexes were recorded in DMSO over 200–500 nm (Fig. 8). Both the free ligand (L) and its complexes show a strong band at approx 345–350 nm, due to π → π* transitions in the aromatic part of the sensitizer. The similar peak positions in the spectra of the complexes indicate that the coordination environment around the metal ion exerts negligible influence on the singlet excited state of L. However, the spectra of the complexes show hypochromic shifts compared to the free ligand. The observed absorption shifts are therefore interpreted primarily in terms of solvent and coordination effects, including differential stabilization of the electronic states and subtle reorganization of the electronic density of the antenna ligand upon metal–ligand interaction. These effects can modify the energies of the π → π* transitions and result in the observed spectral shifts.

**Fig. 8:**
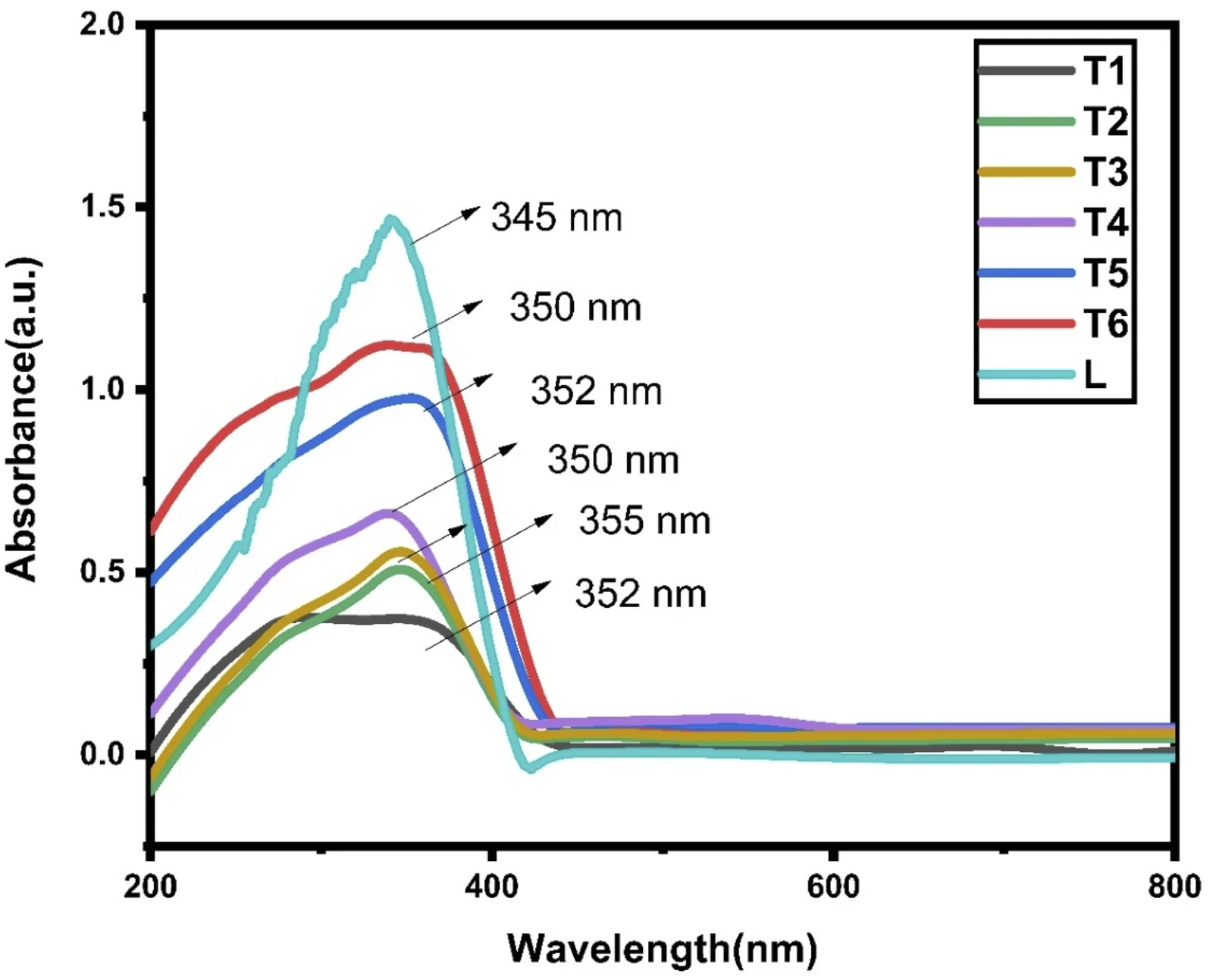
UV-visible spectra of ligand L and its corresponding complexes T1–T6.

To examine the coordination environment, covalency, and symmetry around the metal centers in the complexes, near-infrared (NIR) absorption spectra were recorded for both metal ions. Sm^3+^ transitions are observed primarily in 900–1600 nm, while Tb^3+^ transitions dominate 1600–2900 nm. The Judd–Ofelt analysis was performed separately for the Sm^3+^ and Tb^3+^ emitting centres using their respective electronic transitions. Independent Judd–Ofelt intensity parameters were determined for each lanthanide ion. The data were analyzed using Judd–Ofelt theory, which yields three intensity parameters—*Ω*_2_, *Ω*_4_, and *Ω*_6_—that provide insights into the electronic surroundings of the lanthanide ions. Specifically, *Ω*_2_ indicates the extent of covalent bonding between the metal ion and ligand, *Ω*_4_ relates to long-range electrostatic interactions, and *Ω*_6_ reflects the rigidity of the coordination sphere.

The near-infrared (NIR) absorption spectra of the complexes feature distinct bands at 947, 1088, 1250, 1393, 1501 for Sm^3+^ metal ion with electronic transitions from the 6H_*j*/2_ → 6F_*j*/2_ (*j* = 11, 9, 7, 5, 1) and 1806, 1937, 2184, and 2804 nm for Tb^3+^ metal ion with electronic transitions from ^7^F_6_ → ^7^F_*n*_ (*n* = 1, 2, 3, 4).^38^ The absorption transitions of Sm^3+^ and Tb^3+^ observed in the NIR region are presented in Fig. S5 and S6 respectively. The experimental oscillator strengths were calculated from absorption data using suitable theoretical methods.^39^11
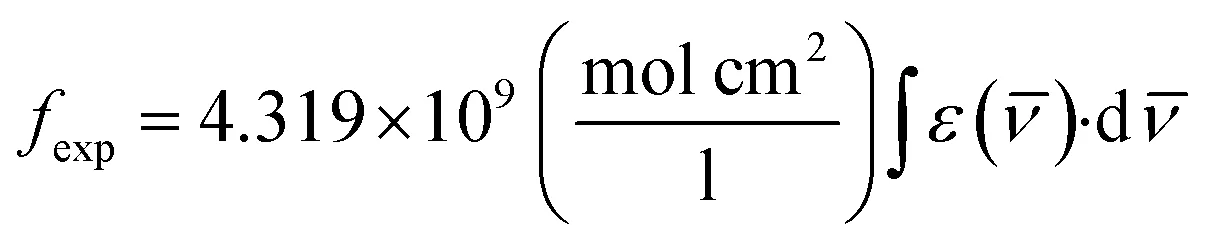


The molar extinction coefficient, *ε*(*ν*), was expressed as a function of wavenumber (cm^−1^) and used following established methods.^40,41^ The analysis focused solely on electric dipole transitions, as magnetic dipole contributions were deemed negligible under the current experimental conditions. Using the Judd–Ofelt (J–O) theoretical framework, the oscillator strength (*f*_cal_) for transitions from the ground state (*ψ*_*J*_) to an excited state (*ψ*′*J*′) was calculated using the standard electric dipole transition formalism, as outlined below:12
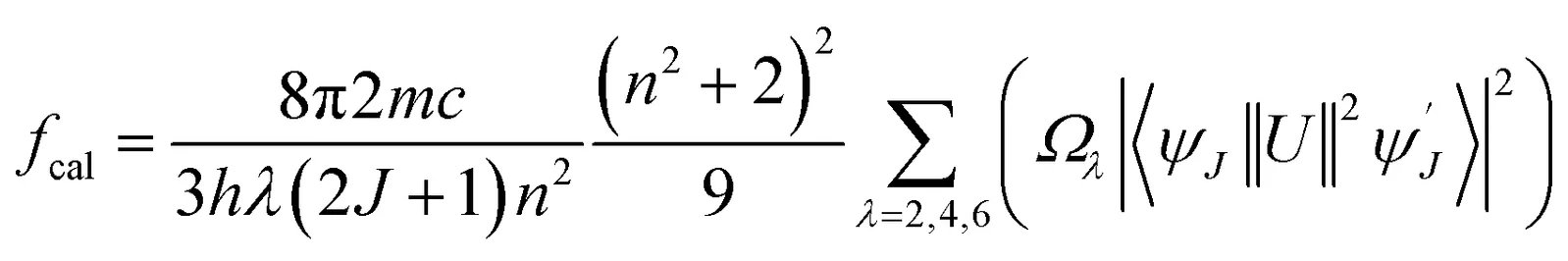


In this context, the constants *c*, *m*, and *h* stand for the speed of light, electron mass, and Planck's constant, respectively. *J* represents the total angular momentum quantum number of the initial state, while *ν* indicates the absorption frequency. The refractive index of the medium is given by *n*. The experimental and calculated oscillator strengths of the Sm^3+^ and Tb^3+^ centres are summarized separately in Tables S1 and S2, respectively.

The Judd–Ofelt intensity parameters, *Ω*_*λ*_ (*λ* = 2, 4, 6), measure the strength of electric dipole transitions. The squared reduced matrix element, 
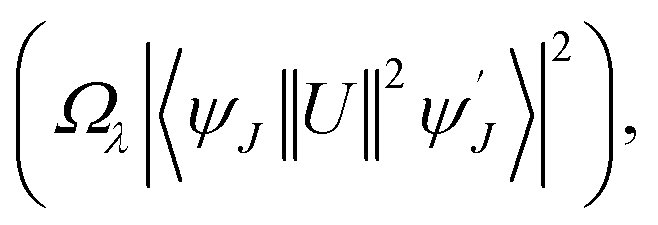
 reflects the transition probability and is independent of the surrounding chemical environment. The *Ω*_*λ*_ parameters were determined by equating the theoretical oscillator strengths (*f*_cal_) with the experimental values (*f*_exp_), as shown in the following equation.13
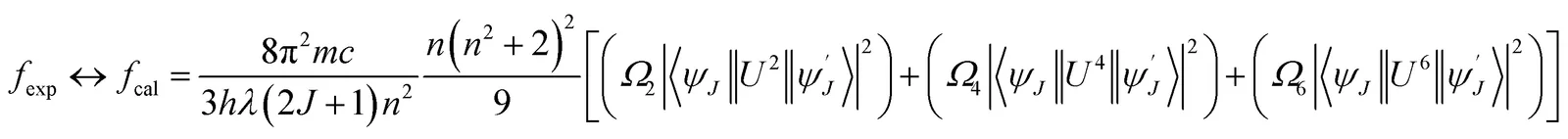


The J–O intensity parameters were determined by least-squares fitting of equations derived from experimentally measured oscillator strengths. For both metal ions, the Judd–Ofelt parameters follow the sequence *Ω*_2_ > *Ω*_4_ > *Ω*_6_. The particularly high value of *Ω*_2_ signifies a strong covalent interaction between the central metal ions and surrounding ligands, pointing to notable 4f–ligand orbital mixing. Conversely, the moderate values of *Ω*_4_ and *Ω*_6_ suggest a relatively rigid coordination geometry, which can affect vibrational coupling and non-radiative relaxation processes. To assess the accuracy of the fitting process, the root-mean-square deviation (*δ*_rms_) was used to measure the difference between the experimental (*f*_exp_) and theoretical (*f*_cal_) oscillator strengths. This statistic was calculated using the following formula.^42^14
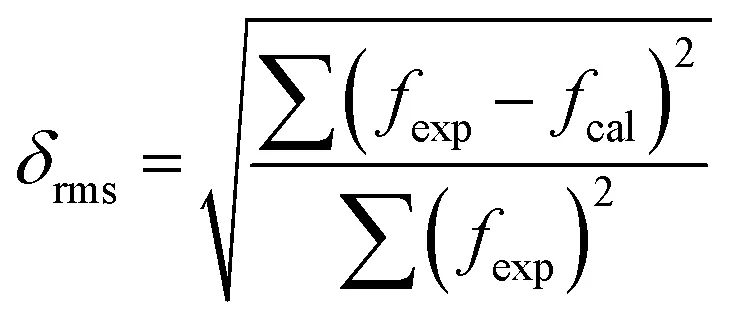


The accuracy of the fitting procedure was evaluated by calculating the root mean square deviation (*δ*_rms_) between the experimental (*f*_exp_) and theoretical (*f*_cal_) oscillator strengths. A lower *δ*_rms_ value reflects a closer correspondence between the observed and predicted data, indicating a more reliable fit. The calculated Judd–Ofelt parameters and the root-mean-square deviation (*δ*_rms_) of the Sm^3+^ and Tb^3+^ centres are summarized separately in Tables S1 and S2, respectively.

### 3.10 Radiative properties

The radiative parameters were calculated independently for Sm^3+^ and Tb^3+^, while the overall radiative contribution of the mixed-lanthanide system was obtained by considering the radiative contributions from both emitting centres. The Judd–Ofelt intensity parameters were used to assess important photophysical properties, such as radiative transition probabilities, total radiative decay rates, non-radiative decay rates, and overall quantum efficiencies. The radiative transition probability from an excited state to a lower energy level was calculated using the following method:15
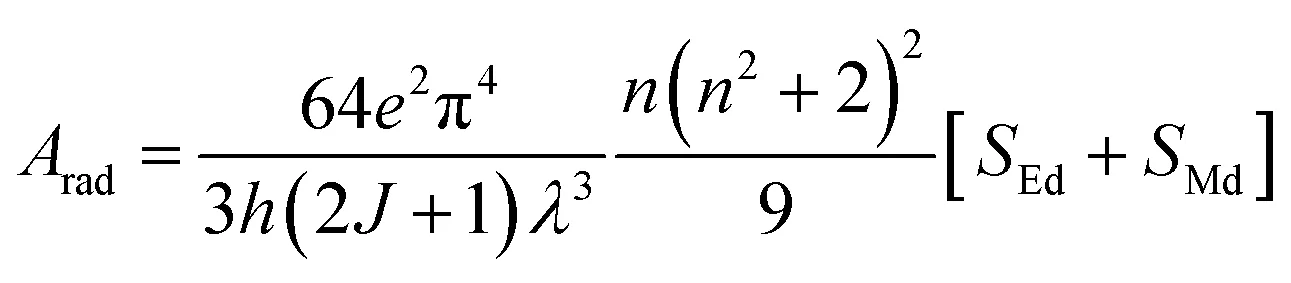


In this context, *S*_Ed_ and *S*_Md_ represent the line strengths associated with electric- and magnetic-dipole transitions, respectively. By neglecting the magnetic dipole contribution, the equation reduces to16
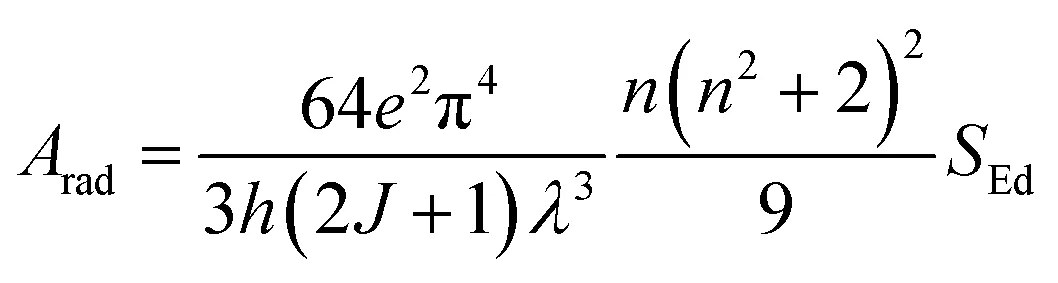
Here, *S*_Ed_ is characterized by Judd–Ofelt parameters and the reduced squared matrix elements.17
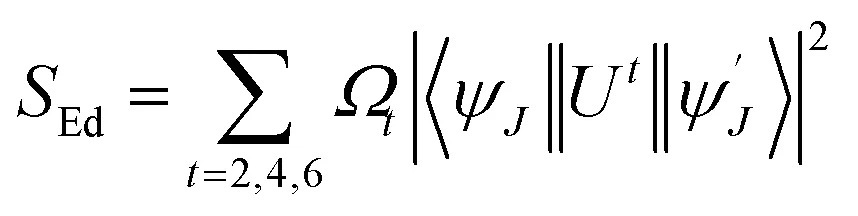


The matrix element values used to calculate the electric dipole line strength were obtained. Using the equation, the radiative transition probabilities for all four emission transitions were calculated, and the total radiative transition probability, *A*_rad_, was calculated by summing these individual values. This total *A*_rad_ was then used to determine other key radiative properties of the complexes.

Lifetime measurements were performed at the prominent emission peak (548 nm) with excitation at 370 nm, using a PL spectrophotometer. As shown in Fig. 9, the decay of the synthetic complexes follows a single-exponential function. The lifetime values were then determined using the following equation.18
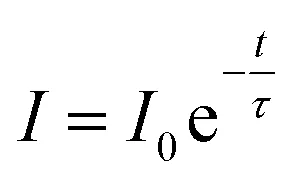
Here, *τ* denotes the luminescence decay time, and *I*_0_ and I are the emission intensities at *t* = 0 and at time *t*, respectively. The lifetime measurements of a single emissive Tb^3+^ center are shown in inset of Fig. 9 and the experimentally derived lifetime value for the corresponding complex is 1.256 ms.

**Fig. 9:**
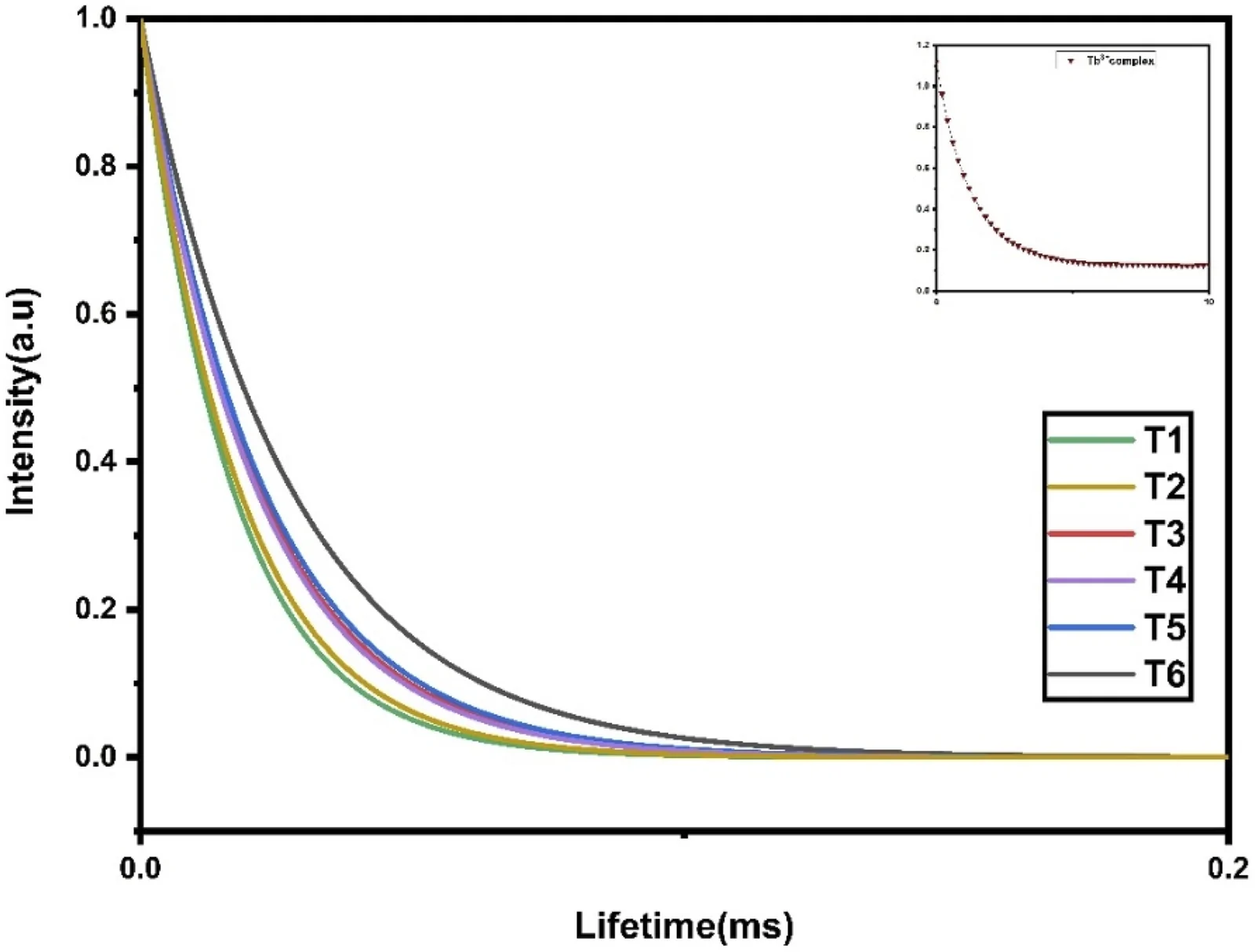
Luminescence decay curves of complexes T1–T6 were performed at the prominent emission peak 548 nm with excitation at 370 nm with inset of Tb^3+^ complex.

The total transition rate is related to the measured decay time by the following expression19
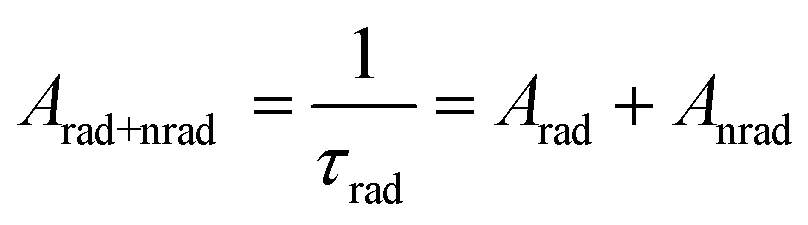


Quantum efficiency (*Q*_eff_) was determined using the following relation and used to evaluate the luminescence performance of the complexes.20
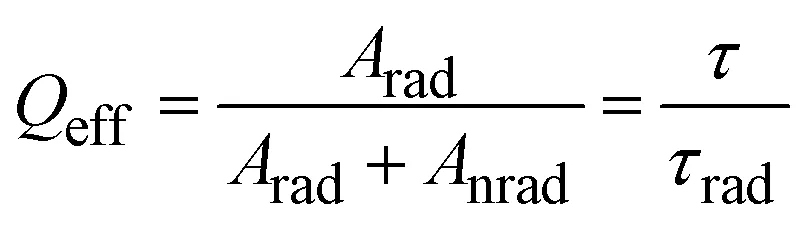


The radiative transition probabilities, radiative lifetimes, non-radiative contributions, and calculated quantum efficiencies are summarized in Table 4. The observed quantum efficiency is influenced by the radiative and non-radiative relaxation pathways. The presence of seven water molecules identified from the TGA analysis may contribute to non-radiative relaxation through high-energy O–H vibrational modes. In addition, the efficiency of ligand-to-metal energy transfer depends on the energetic matching between the ligand excited states and the emitting levels of Sm^3+^ and Tb^3+^. These factors may contribute to the remaining non-radiative losses. Reported lanthanide complexes exhibit a broad range of quantum efficiencies depending on the ligand structure and coordination environment. For example, a quantum yield of 14.3% has been reported for a luminescent lanthanide complex containing Sm/Tb-related systems,^43^ which is comparable to the value obtained in the present work. Furthermore, energy-transfer efficiency was determined using the following equation21
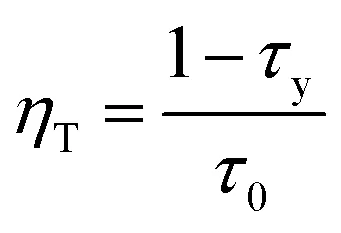
Here, *τ*_0_ and *τ*_y_ denote the lifetimes of Tb^3+^ in the absence and presence of Sm^3+^, respectively. The calculated efficiency for the complexes is summarized in Table 4. A pronounced and systematic reduction in the donor decay lifetime is observed across the series from T6 to T1, decreasing continuously. This substantial shortening of the Tb^3+^ excited-state lifetime provides compelling evidence for progressively enhanced non-radiative quenching. Correspondingly, the calculated energy-transfer efficiency exhibits a remarkable and consistent increase, rising from 71.35% for T6 to a maximum of 80.13% for T1. In addition, the efficiency of ligand-to-lanthanide ion energy transfer depends on the energetic relationship between the ligand excited states and the emissive levels of the lanthanide ions^44^

**Table 4:** Presents the Judd–Ofelt (JO) intensity parameters for T1–T6

	*A*_rad_	*A*_rad+nrad_	*τ* (ms), adjacent *R*^2^	*Q*_eff_	*η*_T_
T1	610.20	4095.00	0.2442 (0.9847)	15.14	80.13
T2	580.15	3700.96	0.2702 (0.9634)	15.07	78.55
T3	565.70	3359.08	0.2977 (0.9785)	16.84	76.77
T4	542.62	3239.39	0.3087 (0.9921)	16.75	75.42
T5	535.10	3218.53	0.3107 (0.9501)	16.62	75.26
T6	410.20	2794.85	0.3578 (0.9714)	16.11	71.35

### 3.11 Energy transfer

When exposed to UV light, the complexes absorb energy, become excited, and then emit characteristic luminescence. To understand the energy-transfer pathway, the singlet and triplet states of both the main ligand (L) and the auxiliary ligand were examined. The singlet energy of L was obtained from its UV-visible absorption edge, while the triplet energy was determined from the phosphorescence spectrum of its Gd-based complex. The triplet level of L (22 421 cm^−1^) is below the Gd^3+^ transition (^6^P_7/2_ → ^8^S_7/2_ at 32 051 cm^−1^), suggesting that L does not effectively sensitize Gd^3+^. As a result, the Gd^3+^ complex shows only ligand-centered phosphorescence, consistent with similar ligand systems described in the literature.^22^ The energies of the singlet and triplet states of L match reported values, and the auxiliary ligand offers additional excited-state pathways that improve energy-transfer efficiency.

The proposed mechanism starts with UV excitation of the ligands to the singlet state (S_1_), then involves intersystem crossing to the triplet state (T_1_). Energy is subsequently transferred to the levels of Sm^3+^ and Tb^3+^. From the T_1_ level, energy can either transfer to Sm^3+^*via* quadrupole–quadrupole coupling or decay radiatively to the ground states (^7^F_6_, ^7^F_5_, ^7^F_4_). The radiative decay of Sm^3+^ level to its ground states (^6^H_5/2_, ^6^H_7/2_, ^6^H_9/2_). The energy transfer pathway depicted in Fig. 10. The cooperative energy transfer between Tb^3+^ and Sm^3+^ enables precise color tuning, as shown in the CIE diagram (Fig. 7), with a smooth transition from yellow to near-white emission by adjusting ion ratios.

**Fig. 10:**
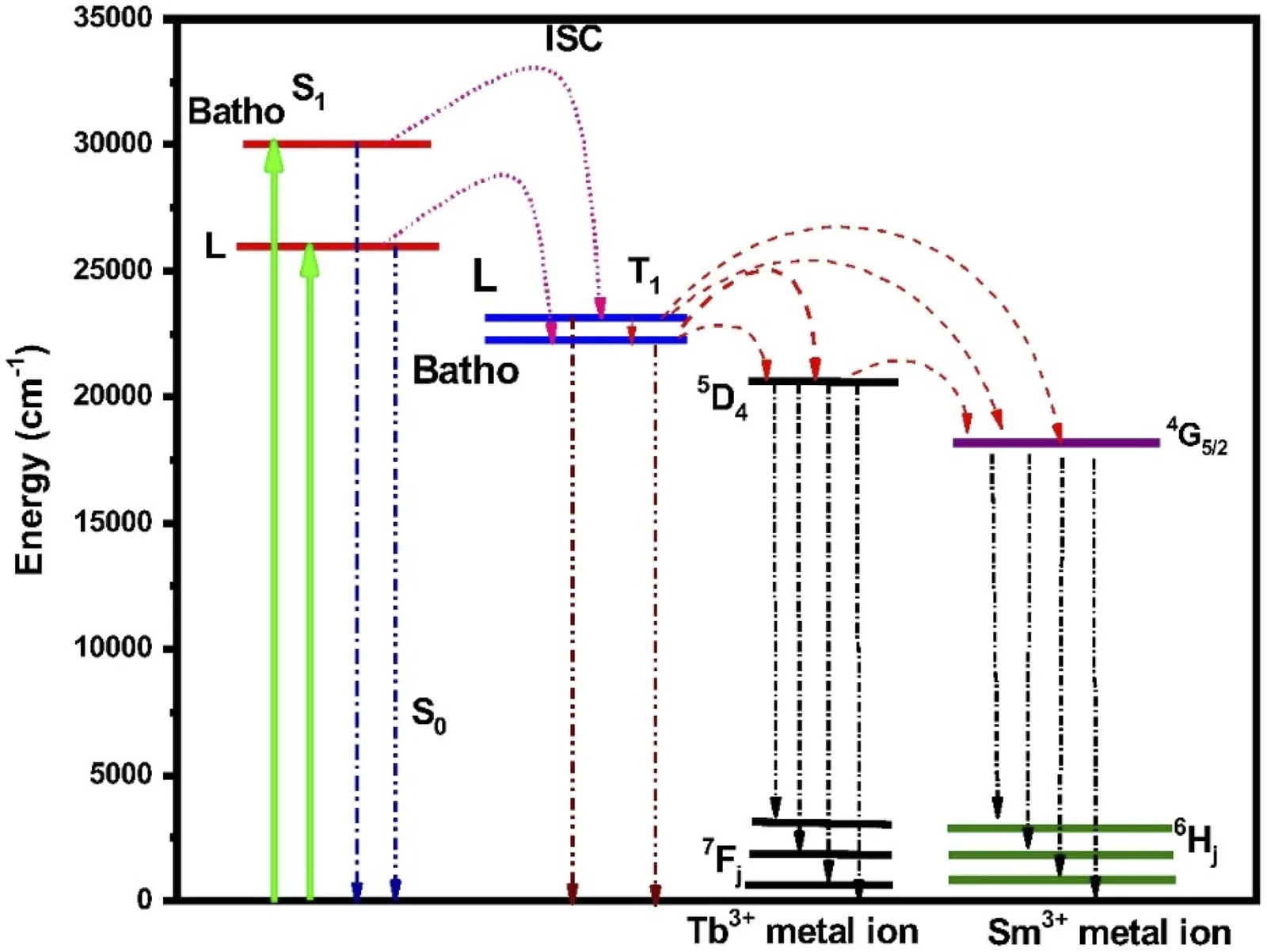
Energy transfer pathway for complexes.

## 4 Conclusion

This study shows that the color-tunable [Tb_*X*_Sm_1−*X*_·L_3_·batho] mixed complexes, synthesized *via* solution precipitation method. By adjusting their composition, these complexes emit near-white light possess desirable semiconductor properties. Extensive characterization, including elemental analysis, PXRD, SEM, IR, UV-vis, TGA-DTG, and photoluminescence spectroscopy, confirmed the structural integrity and multifunctional nature of the complex. Thermogravimetric analysis demonstrated excellent thermal stability up to 255 °C, validating their suitability as emitting materials for OLED fabrication. The estimated band gap energy confirms the semiconducting nature of the complexes, and the corresponding electrochemical measurements provide complementary information about the electronic structure of the material. Band gap and Urbach energy values further highlighted their potential in solar cells and semiconductor applications. Analysis of the Judd–Ofelt phenomenological parameters (*Ω*_2_ > *Ω*_4_ > *Ω*_6_) provided valuable insight into the asymmetry and rigidity of the coordination environment, offering a comprehensive understanding of their photophysical behavior. Their capacity to reveal latent fingerprints demonstrates a unique forensic application, broadening their relevance to security and analysis. These results underscore the promise of such complexes for next-generation optoelectronic application and highlight their relevance in advancing adaptive luminescent materials for applications in lighting, display technologies, and photonics.

## Author contributions

Bhawna Rathee: investigation, conceptualization, data curtain, formal analysis, writing – original draft. Vaishnavi Lather: contributed to an investigation. Rajesh Kumar: supervision, formal analysis, writing – review & editing.

## Conflicts of interest

The authors say there are no conflicts of interest regarding this research work.

## Supplementary Material

RA-OLF-D6RA07242A-s001

## Data Availability

The data supporting the findings of this study are available within the article and its supplementary information (SI). Supplementary information (SI) is available. See DOI: https://doi.org/10.1039/d6ra07242a.
