# Supplementary material for: Designing bimetallic lanthanide complexes with composition controlled white-light luminescence for advanced optoelectronics
Source: RSC Adv. 2026 Sep 29. Online ahead of print. doi: 10.1039/d6ra07242a (PMC13621421; doi:10.1039/d6ra07242a)
Supplement: Supplementary file 1 [file RA-OLF-D6RA07242A-s001.pdf]

Supplementary Information (SI) for RSC Advances.
This journal is © The Royal Society of Chemistry 2026
Supplementary Information (SI) for RSC Advances.
This journal is © The Royal Society of Chemistry

Designing Bimetallic Lanthanide Complexes with Composition Controlled White-Light Luminescence for Advanced Optoelectronics

Bhawna Rathee[a], Vaishnavi Lather[b], and Rajesh Kumar[a]*

[a]University Institute of Engineering and Technology, Maharshi Dayanand University, Rohtak - 124001, India

[b]Shri Guru Ram Rai Institute of Medical and Health Sciences, Dehradun - 248001, India

*Corresponding author: E-mail address: lather_rajesh@yahoo.com (Rajesh Kumar)

E-mail address of authors: bhawnarathee7@gmail.com, vaishnavilather990@gmail.com

Latent fingerprint revelation:

Latent fingerprint detection remains a crucial technique in forensic investigations, where clearly visualizing ridge details is vital for accurate personal identification. Despite ongoing advancements, producing high-contrast, well-defined fingerprint patterns on various surfaces remains challenging. As a result, there is increasing interest in developing advanced luminescent materials to enhance sensitivity, resolution, and reliability in fingerprint visualization. Traditional powders, including metallic formulas and fluorescent dyes like rhodamine-based materials, have been widely used due to their effective imaging. However, these agents often have limitations, including potential toxicity, health risks from repeated exposure, respiratory irritation, carcinogenic concerns associated with certain dyes, and contamination risks during routine handling. These issues have led to the search for safer, more effective alternatives. Lanthanide-based luminescent materials are promising due to their excellent photostability, chemical stability, low toxicity, and narrow-band emissions. Notably, Sm^{3+} and Tb^{3+} containing complexes emit intense, high-contrast fluorescence under UV light, allowing selective visualization of latent fingerprints with minimal background noise. Their superior optical properties, along with environmental and biological safety, make them ideal candidates for advanced forensic imaging. Building on these benefits, this study presents the use of the T6 complex as a luminescent powder to develop latent fingerprints on glass surfaces, producing clear ridge details under UV light using gel documentation software and demonstrating its potential for practical forensic use.

Latent fingerprints deposited at different residue densities were effectively developed using the synthesized complex, producing sharp, high-contrast ridge patterns even when only faint fingerprint residue was present. This observation demonstrates the excellent sensitivity of the material toward trace fingerprint constituents. The luminescent images remained stable during continuous exposure to 370 nm ultraviolet radiation, confirming the superior photostability of the complex under the selected excitation conditions. In contrast, exposure to shorter-wavelength UV light resulted in a noticeable reduction in emission intensity, indicating that 370 nm excitation provides the most suitable balance between luminescence efficiency and imaging stability.

Forensic fingerprint visualization relies on three levels of identifying characteristics. The first level consists of the overall ridge flow and pattern types, including arches, loops, and whorls.

The second level comprises individual ridge minutiae, such as ridge endings, bifurcations, bridges, and enclosed ridges, which serve as the principal features for fingerprint comparison and identification. The third level encompasses microscopic details, including sweat pores and ridge edge morphology, providing highly distinctive information for individualization.

Upon UV excitation, the T6 complex generated white luminescence, consistent with its CIE chromaticity coordinates, enabling clear visualization of fingerprint impressions. As illustrated in Fig S4, the developed fingerprints revealed well-resolved Level 1, Level 2, and Level 3 qualitative characteristics, demonstrating exceptional image clarity and ridge definition. These findings underscore the effectiveness of the lanthanide-based luminescent complex as a highly sensitive, photostable, and reliable material for latent fingerprint development, highlighting its promising potential for practical forensic applications.

Table S1 Judd–Ofelt parameters, experimental and calculated oscillator strengths, and δ_{rms} for Sm^{3+} ion of the complexes.

**Transitions**	**T1**		**T2**		**T3**		**T4**		**T5**		**T6**	
	**f_{exp} (10^{-5})**	**f_{cal}**	**f_{exp}**	**f_{cal}**	**f_{exp}**	**f_{cal}**	**f_{exp}**	**f_{cal}**	**f_{exp}**	**f_{cal}**	**f_{exp}**	**f_{cal}**
**$^6H_{5/2}\rightarrow{}^6F_{11/2}$**	0.1010	0.1034	0.1430	0.1487	0.1200	0.1270	0.1350	0.01375	0.1400	0.1423	0.1200	0.1255
**$^6H_{5/2}\rightarrow{}^6F_{9/2}$**	0.0641	0.0489	0.1240	0.0880	0.2020	0.1579	0.0650	0.0493	0.0618	0.0472	0.1800	0.1456
**$^6H_{5/2}\rightarrow{}^6F_{7/2}$**	0.0185	0.0500	0.0591	0.1350	0.0425	0.1365	0.0150	0.0477	0.0132	0.0435	0.0520	0.1241
**$^6H_{5/2}\rightarrow{}^6F_{5/2}$**	0.0562	0.0321	0.1610	0.0963	0.1540	0.0672	0.0580	0.0321	0.0520	0.0294	0.1200	0.0609
**$^6H_{5/2}\rightarrow{}^6F_{1/2}$**	0.651	0.0052	0.1220	0.0160	0.1030	0.0100	0.0624	0.0053	0.0660	0.0049	0.1230	0.0090
**$\Omega_2\times10^{-19}$**	0.3315		0.4767		0.4071		0.4407		0.4562		0.4022	
**$\Omega_4\times10^{-19}$**	0.5990		0.1216		0.2703		0.0480		0.0419		0.2462	
**$\Omega_6\times10^{-19}$**	0.0385		0.1190		0.0718		0.0392		0.0361		0.0650	
**$\delta_{rms}\times10^{-5}$**	0.494		0.529		0.543		0.4186		0.418		0.537	

Table S2 Judd–Ofelt parameters, experimental and calculated oscillator strengths, and δ_{rms} for Tb^{3+} ion of the complexes.

Transitions	T1		T2		T3		T4		T5		T6	
	f_{exp} (10^{-6})	f_{cal}	f_{exp}	f_{cal}	f_{exp}	f_{cal}	f_{exp}	f_{cal}	f_{exp}	f_{cal}	f_{exp}	f_{cal}
$^7F_6 \rightarrow {}^7F_1$	0.1100	0.3488	0.1300	0.3387	0.1120	0.3402	0.1280	0.3422	0.1170	0.3447	0.1300	0.3621
$^7F_6 \rightarrow {}^7F_2$	0.7100	0.4684	0.6700	0.4572	0.6950	0.4622	0.6840	0.4655	0.6970	0.4647	0.7200	0.4832
$^7F_6 \rightarrow {}^7F_3$	0.4910	0.5465	0.5010	0.5495	0.5120	0.5650	0.5210	0.5708	0.5040	0.5569	0.5090	0.5629
$^7F_6 \rightarrow {}^7F_4$	0.5890	0.5890	0.6020	0.6020	0.6175	0.6175	0.6290	0.6290	0.6000	0.6000	0.5895	0.5895
$\Omega_2 \times 10^{-19}$	0.2398		0.2311		0.2082		0.2206		0.2100		0.2069	
$\Omega_4 \times 10^{-19}$	0.1667		0.1769		0.1880		0.1911		0.1782		0.1696	
$\Omega_6 \times 10^{-19}$	0.1550		0.1505		0.1512		0.1521		0.1532		0.1601	
$\delta_{rms} \times 10^{-6}$	0.2350		0.2100		0.2237		0.2088		0.2257		0.2272	

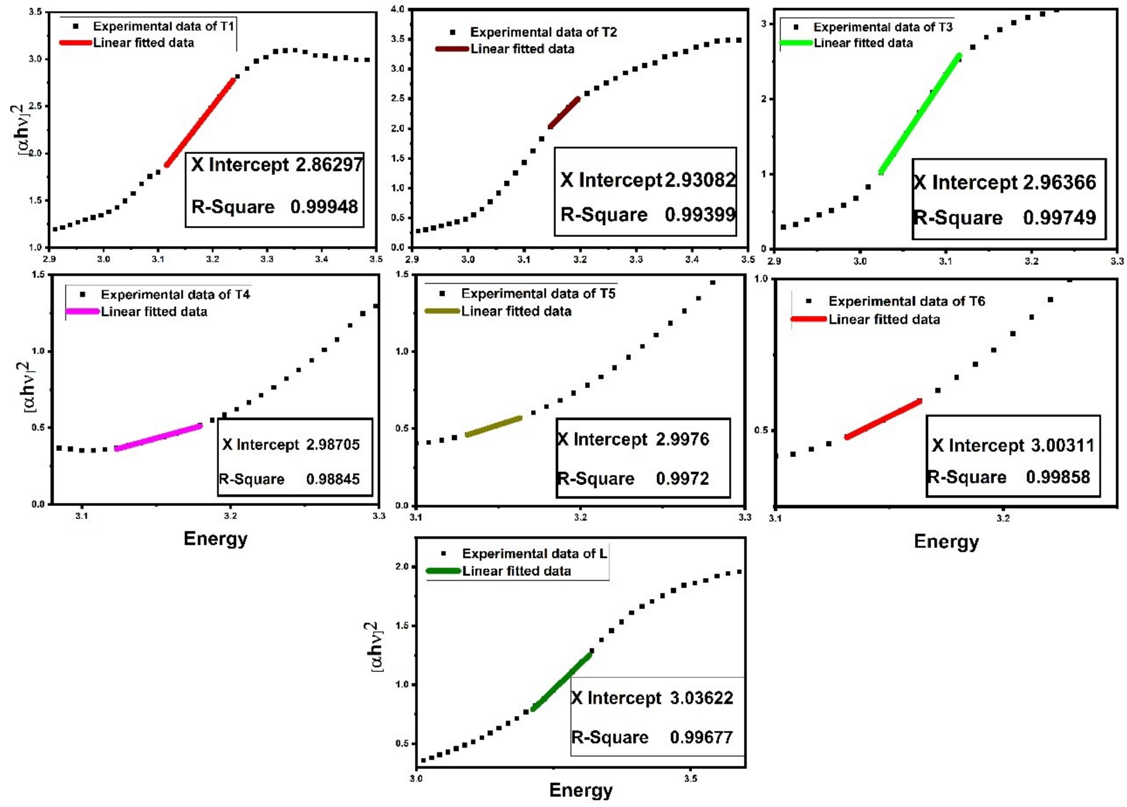

Fig S1: Linearly fitted Tauc's curves of L and Complexes to obtain band gap value.

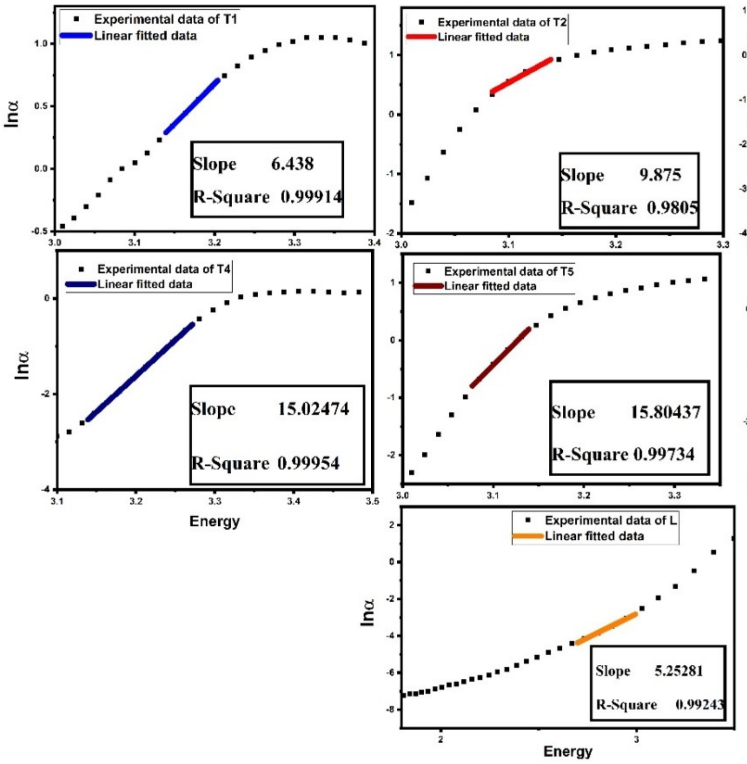

Fig S2: Linear fitting of experimental data of absorption coefficient to obtain the Urbach energy of L and Complexes

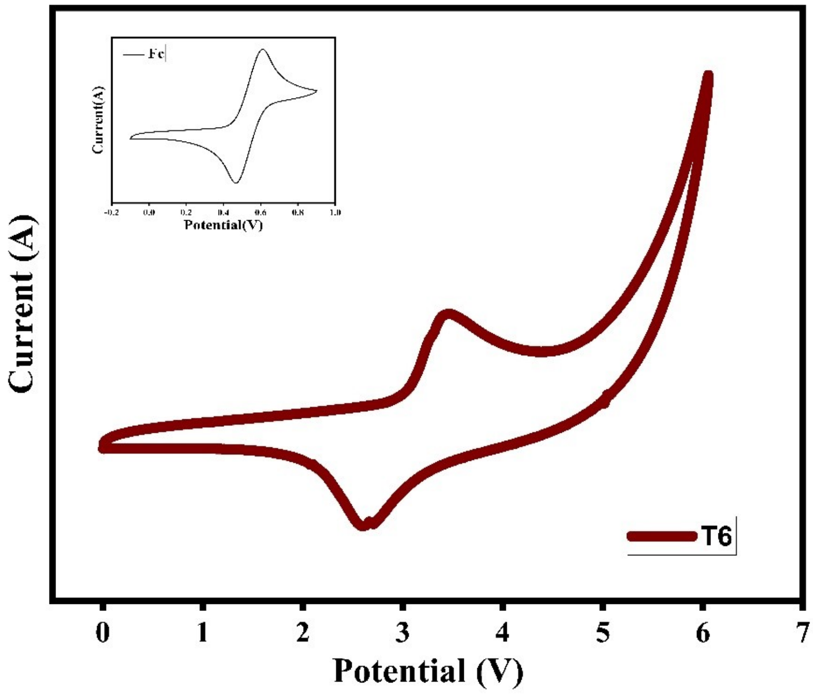

Fig S3. Current–voltage (I–V) characteristics of T6, with ferrocene (Fc).

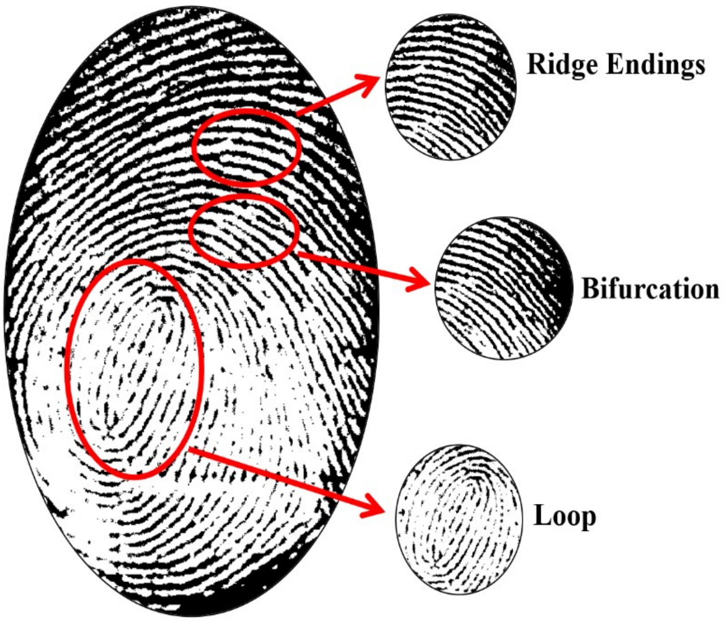

Fig S4. Photographic visualization of latent fingerprints under UV light using gel documentation system.

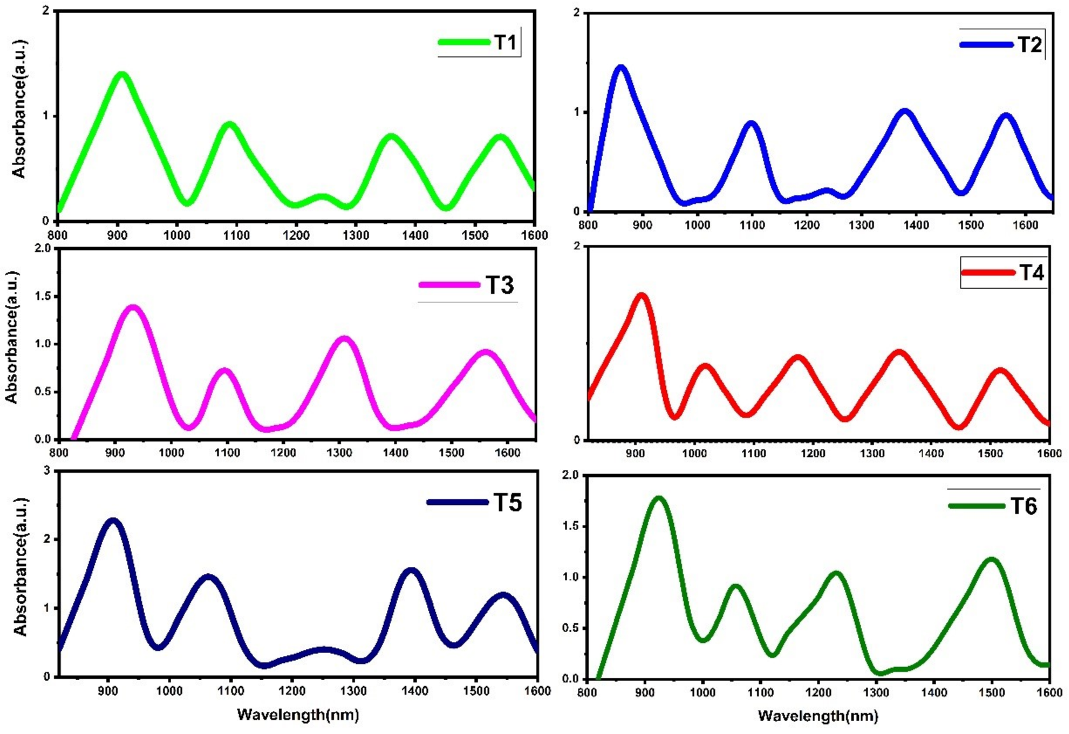

Fig S5. Schematic representation of the 4f–4f electronic transitions of Sm^{3+} ion used for the determination of the Judd–Ofelt parameters.

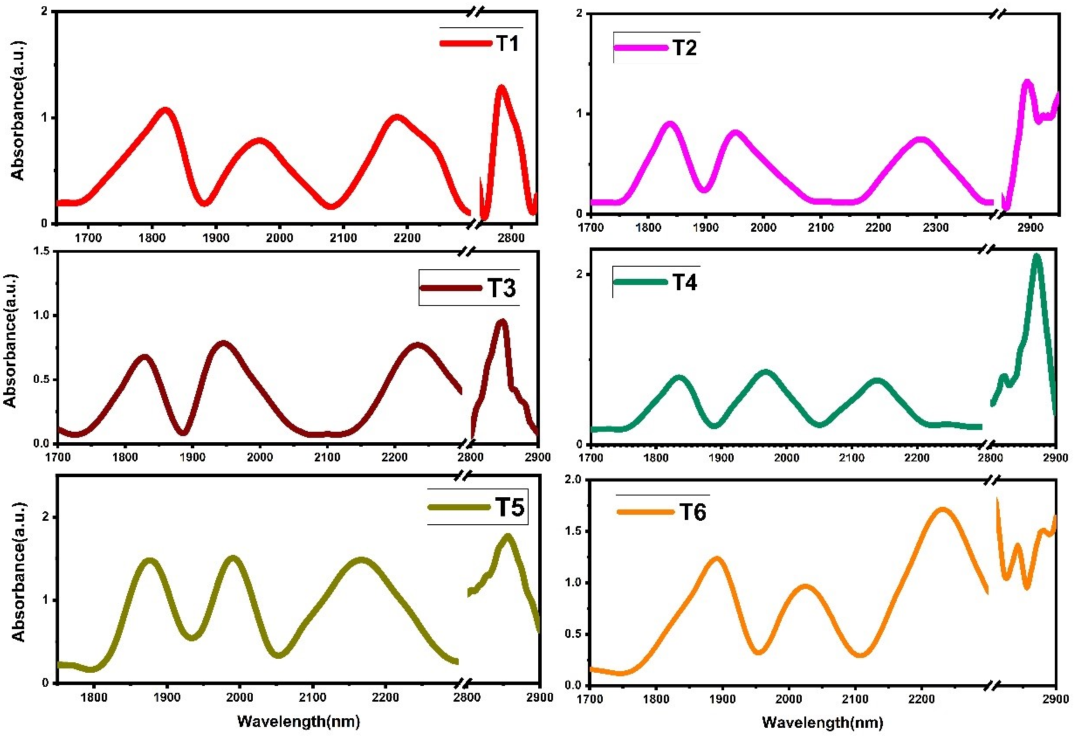

Fig S6. Schematic representation of the 4f–4f electronic transitions of Tb^{3+} ion used for the determination of the Judd–Ofelt parameters.

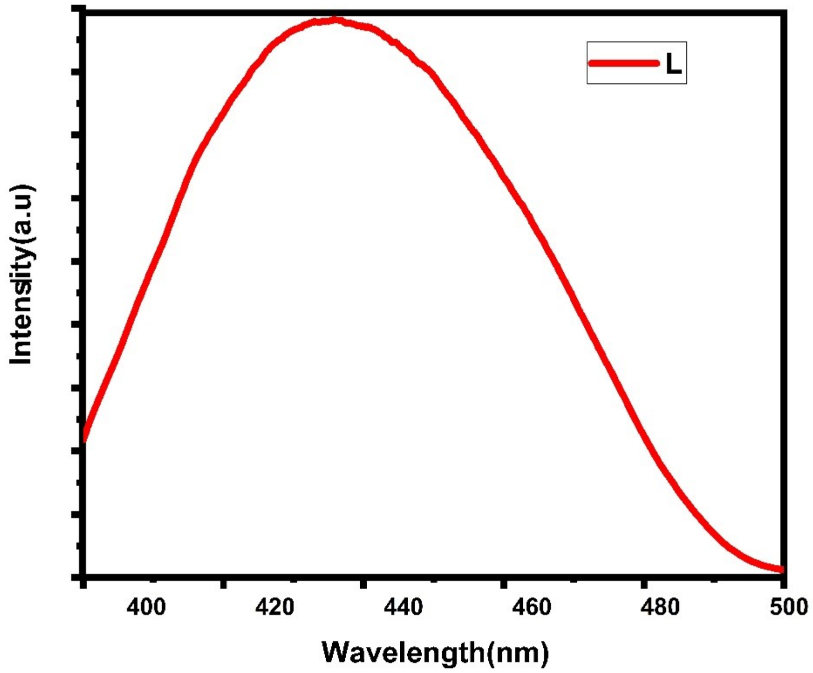

Fig. S7 Solid state emission spectra of primary Ligand (L) recorded at the excitation wavelength of 370 nm.
